# Near-infrared prediction of total phosphorus in leaves content in korla fragrant pear with growth period specificity via spectral modeling

**DOI:** 10.3389/fpls.2025.1666460

**Published:** 2025-10-10

**Authors:** Mingyang Yu, Junkai Zeng, Yang Li, Weifan Fan, Lanfei Wang, Hao Wang, Jianping Bao

**Affiliations:** ^1^ Xinjiang Production and Construction Corps, Tarim University Institute of Horticulture and Forestry, Xinjiang Alar, China; ^2^ Tarim Basin Biological Resources Protection and Utilization Key Laboratory, Xinjiang Production and Construction Corps, Xinjiang Alar, China; ^3^ Xinjiang Production and Construction Corps, Southern Xinjiang Special Fruit Trees High -Quality, High -Quality Cultivation and Deep Processing of Fruit Products Processing Technical National Local Joint Engineering Laboratory, Xinjiang Alar, China; ^4^ Ministry of Education of the People’s Republic of China, Nanjing Agricultural University Horticulture and Forestry College, Nanjing, Jiangsu, China

**Keywords:** korla fragrant pear, total phosphorus in leaves, near-infrared spectrum, growth period specificity, machine learning

## Abstract

Leaf total phosphorus content (LTP) is a key indicator for assessing fruit nutrition status. As a rapid non-destructive inspection method, Near-infrared spectroscopy technology is susceptible to the influence of changes in plant growth periods and spectral noise on its prediction accuracy. At present, how to synergistically utilize growth period information and Spectral pre - processing methods to optimize the LTP Prediction model remains to be further studied. The study systematically collected Leaf sample and their near-infrared Spectral data during three key growth periods of Korla fragrant pear (fruit-setting period, fruit swelling period, and Maturity period). In the Spectral pre-processing stage, multiple scattering correction, Savitzky-Golay Smooth, First Derivative (FD), Second Derivative (SD) and their combined algorithms were comprehensively applied. The Competitive Adaptive Reweighted Sampling (CARS) algorithm was used for characteristic wavelength selection, and based on this, Growth period specificity BP neural network model and cross-growth period general prediction models were constructed respectively to evaluate the performance of different Modeling strategies. Results The study showed that LTP content exhibited a significant differential distribution across different growing stage. In the characteristic wavelength bands, after processing with Combined pre-processing method (e.g., MSC+ FD), the correlation coefficient between the spectrum and LTP content significantly increased to approximately 0.90. The predictive performance of the Growth-period-specific model was comprehensively superior to that of the general model, with the Validation set coefficient of determination remaining above 0.83. Compared with the general model, the Coefficient of determination (R^2^) increased by 0.05-0.16, and the root mean square error decreased by 0.0029-0.0079. This study successfully constructed a technical system of “Growth period-Preprocessing-Model”. The results indicated that the Modeling strategy considering the characteristics of crop growing stage could significantly improve the predictive ability of near-infrared spectroscopy models. This study provides a reliable technical framework for Precision nutrient management in orchard, and the established methodology can also serve as a reference for nutrient Surveillance of other fruit tree plants.

## 1 Introduction

Phosphorus as an essential mineral element for plant growth and development plays a critical role in physiological processes such as nucleic acid synthesis, energy metabolism, and the maintenance of cell membrane structures (Chen et al., 2021; Song et al., 2024).The dynamic change of Leaf total phosphorus (LTP) content is not only a direct reflection of fruit nutrition status, but also an important basis for precise fertilization in orchards (Shah et al., 2024).As a characteristic cash crop in the arid regions of northwestern China, Phosphorus nutritional diagnosis in korla fragrant pear has significant practical implications for improving fruit quality and yield (Wang et al., 2022).

Near-infrared spectroscopy (NIRS) technology offers an innovative approach for *in-situ* monitoring of nutritive element of plant due to its advantages of non-destructive inspection, high-throughput analysis, and rapid response. By capturing the vibrational absorption features of hydrogen-containing groups (e.g., P-O-H), it enables spectrum analysis of leaf Phosphorus content (Murguzur et al., 2019). 

Current research on fruit tree Phosphorus Spectral diagnosis faces three bottlenecks that require breakthroughs: First, most studies have not systematically considered the impact of Growth period differences on leaf Phosphorus distribution. The phosphorus metabolism characteristics of korla fragrant pear differ significantly during the fruit-setting period, fruit-expanding period, and maturity, exhibiting distribution patterns of low content and high dispersion during the fruit-setting period, stable state during the fruit-expanding period, and high content and high dispersion during maturity (**Figure 2**). These patterns necessitate model construction that adapts to the physiological characteristics of different phenological periods. However, existing studies mostly adopt an intertemporal general model, limiting prediction accuracy (Siedliska et al., 2021).Second, the synergistic mechanism of Spectral preprocessing technology remains unclear. Although single preprocessing methods (such as Multivariate scattering correction MSC, Derivative processing FD/SD) can separately achieve physical interference elimination or chemical characteristics enhancement, they struggle to simultaneously meet the dual requirements of noise suppression and dynamic information preservation. Systematic exploration of optimized combined preprocessing strategies is still lacking (Han et al., 2025; Qi et al., 2025).Third, Feature band selection and Model parameter optimization are not dynamically coupled with the Growth period. The spectral absorption peak associated with Phosphorus (4000–7500 cm^-^¹) exhibits significant differences in response intensity across different Growth period, whereas traditional feature selection algorithms fail to fully exploit this time-space specificity, resulting in insufficient model generalization ability (Tian et al., 2024).

**Figure 1:**
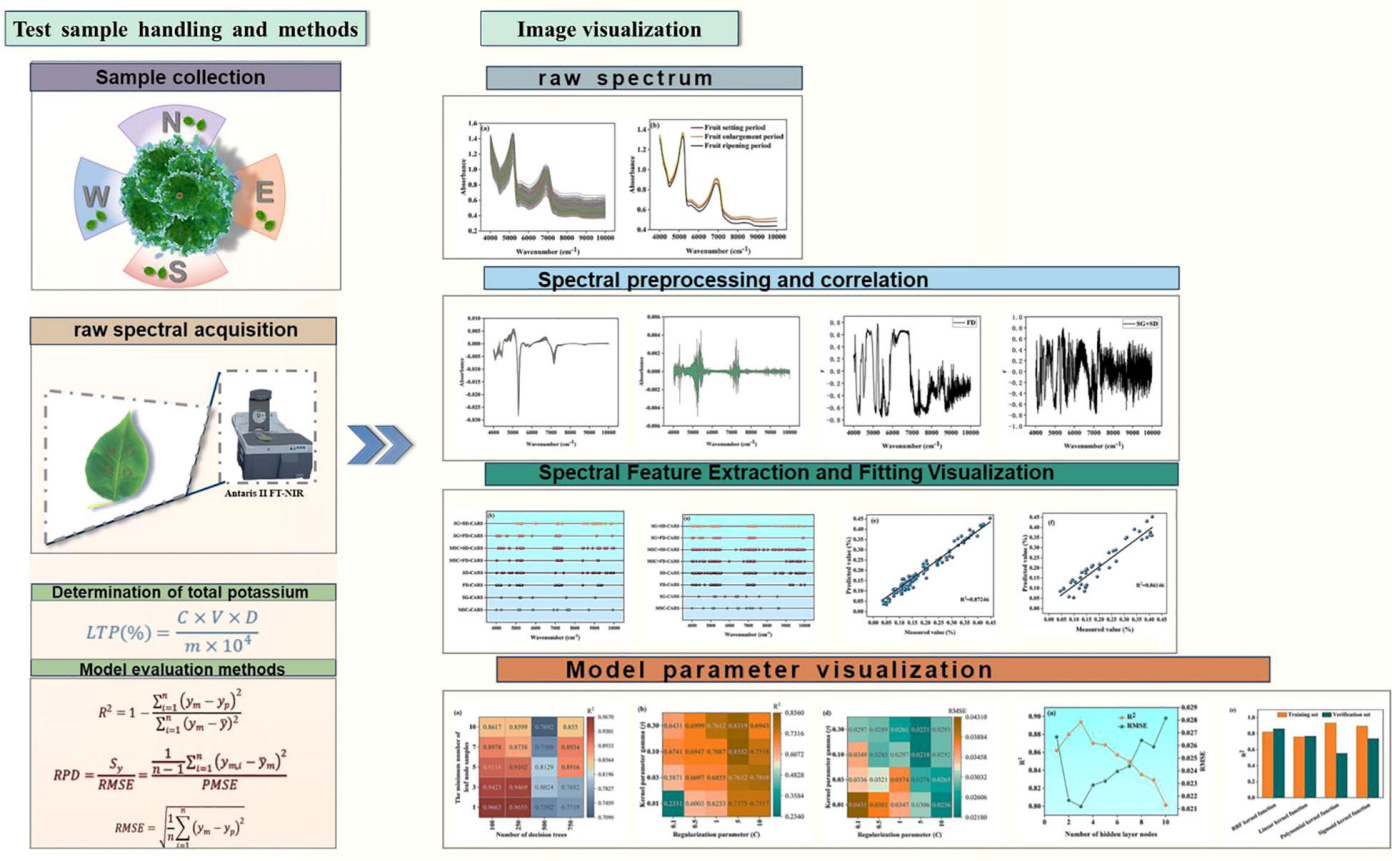
Experimental overall visualization flowchart.

**Figure 2:**
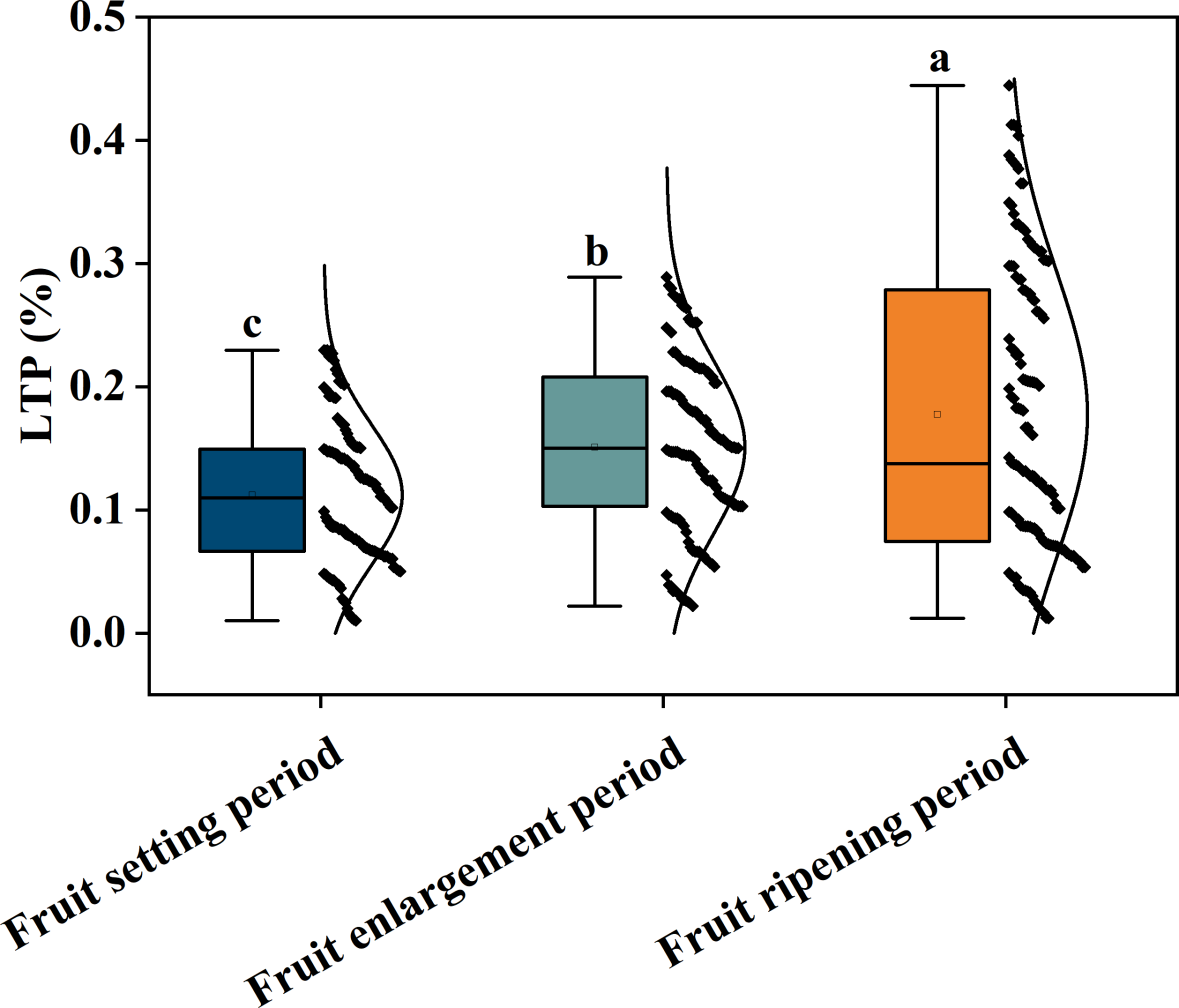
Content of korla fragrant pear total phosphorus in leaves in different periods; Different letters indicate significant differences between groups(P< 0.05).The black dots represent the LTP content of each sample within each Growth period, and the black curve represents the foot normal distribution.

It is worth noting that although near-infrared spectrum analysis has been widely applied in the non-destructive detection of crop nutrients, research on quantitative prediction of phosphorus in fruit trees remains insufficient. Most current studies have focused on field crops such as wheat (Zhang et al., 2022) and rice (Arias et al., 2021), with inadequate exploration of the relationship between leaf total phosphorus (LTP) content and spectral response in fruit tree leaves such as korla fragrant pear. Due to the relatively complex morphological structure of fruit tree leaves, combined with variations in the canopy microenvironment and physiological dynamics at different growing stages, the difficulty of Spectral modeling is increased. Existing research methods often directly apply Traditional regression algorithms such as PLSR and SVR (Ahmadi et al., 2021), failing to conduct targeted model improvement based on the spectral characteristics of fruit trees, and particularly lacking a systematic research approach that integrates phenological change, preprocessing method, and machine learning model. Therefore, establishing a spectral prediction model for LTP content that can respond to the Growth period characteristics of fragrant pear is of great significance for achieving precise monitoring of phosphorus nutrition.

To address the above research bottlenecks, this study aims to overcome the limitations of traditional general models and achieve systematic innovation from theoretical, technical, and applied perspectives, specifically reflected in: 1. Systematically analyzing the unique distribution patterns (left-skewed, stable, right-skewed) of LTP content in korla fragrant pear at different Growth periods (Fonseca-García et al., 2021)and their differential requirements for spectral models, providing a solid physiological basis for Stage-based modeling.2. In-depth exploration of various preprocessing methods (single and combined) under different growth periods within the Collaborative optimization mechanism (e.g., MSC+FD for high-dispersion stages, SG+SD for weak-signal stages), rather than simple stacking, to achieve efficient spectral information purification (Tan et al., 2025). 3. Construction of a complete technical system of “growth period specificity-preprocessing collaboration-model adaptation” to validate the performance improvement of the Stage-based modeling strategy compared to a general model (Cao et al., 2021), providing a directly applicable solution for precision orchard management.

**Figure 3:**
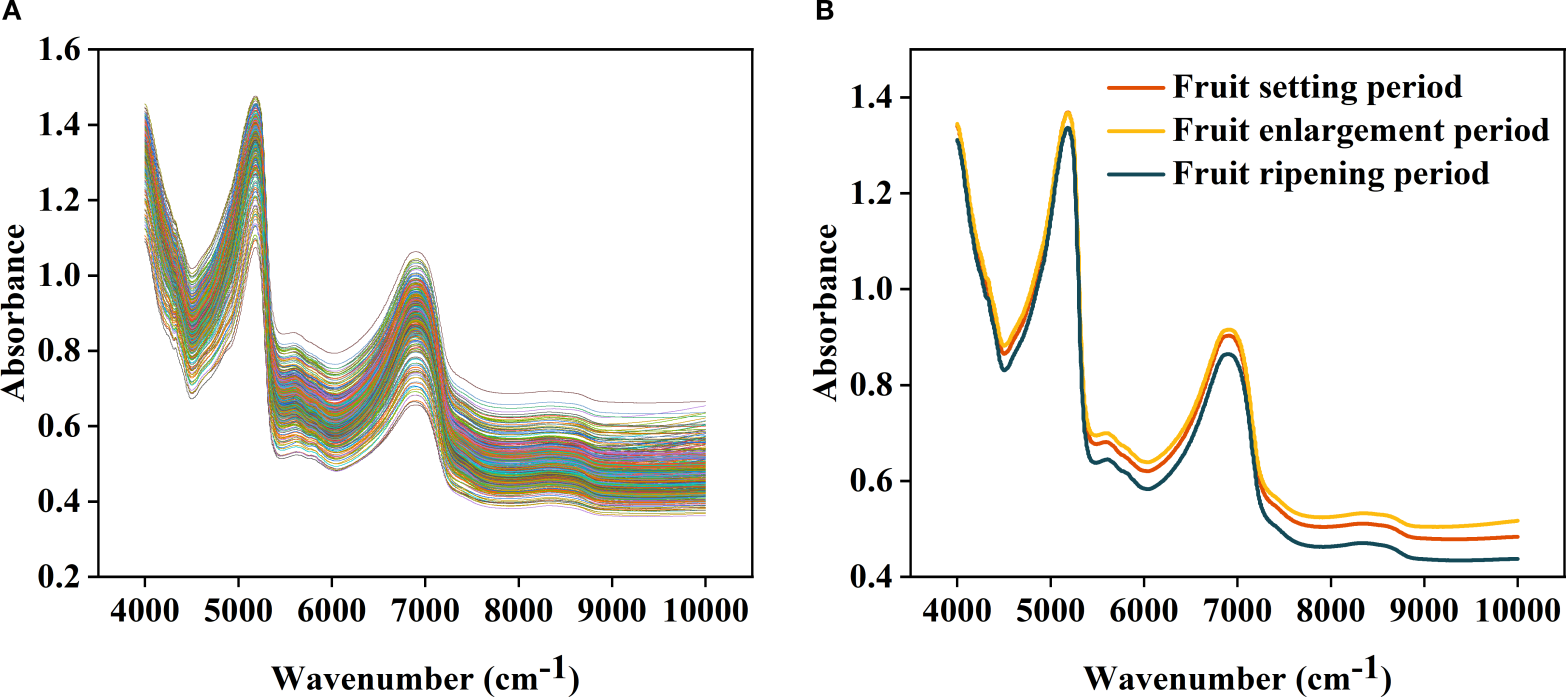
Analysis of original spectral images: **(A)** is the original spectral image; **(B)** is the interior visualization of the average of human spectral images over different periods.

**Figure 4:**
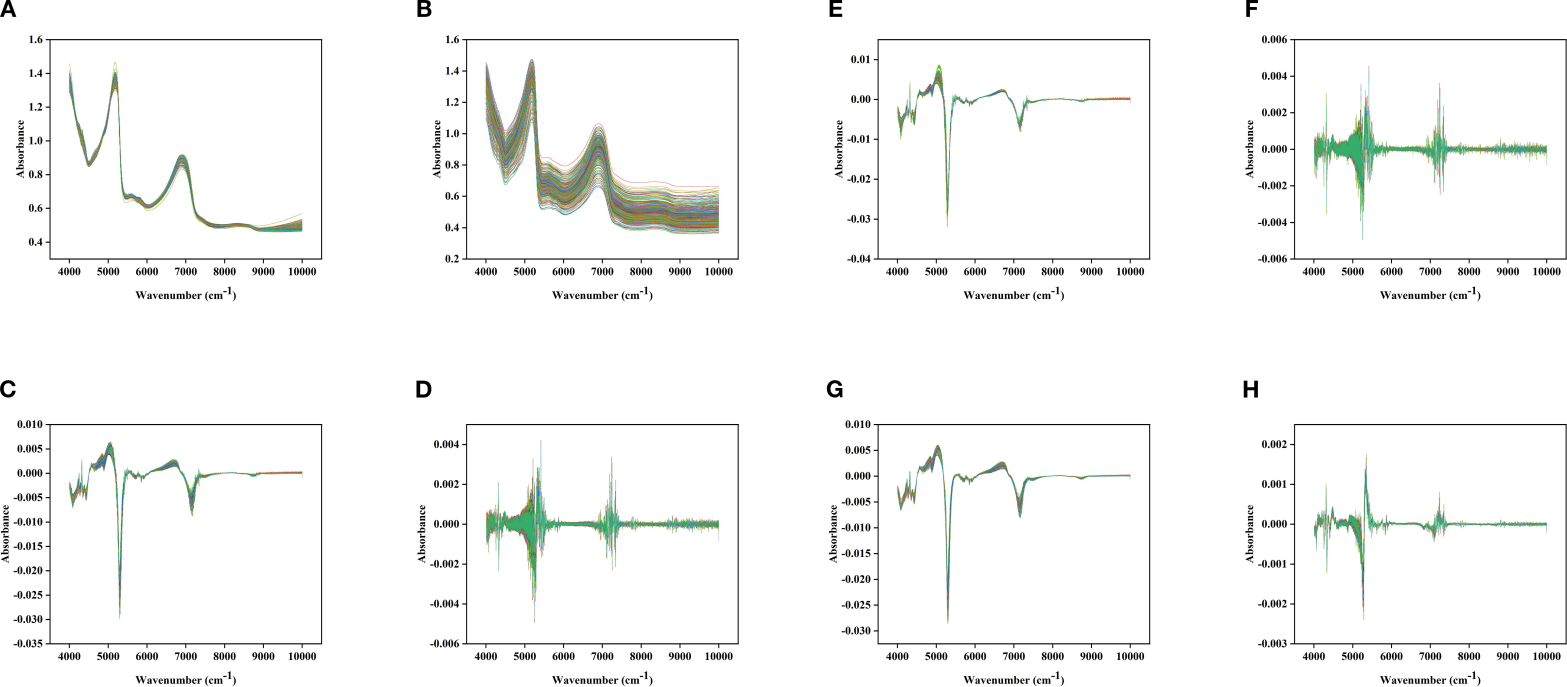
Different spectral images under different preprocessing methods: **(A)**: MSC; **(B)**: SG; **(C)**: FD; **(D)**: SD; **(E)**: MSC+FD; **(F)**: MSC+SD; **(G)**: SG+FD; **(H)**: SG+SD.

To this end, this study first analyzes the distribution characteristics of leaf total phosphorus (LTP) in korla fragrant pear across different growth periods using Box plot, clarifying the content dynamics during the fruit-setting period (minimum 0.02%, maximum 0.25%, left-skewed distribution), fruit-expanding period (median 0.15%, concentrated in 0.10%–0.20%), and maturity period (maximum 0.45%, right-skewed distribution) (**Figure 2**), providing a physiological basis for Spectral modeling; secondly, integrating MSC, SG smoothing, FD/SD derivative processing, and combined strategies (MSC+FD, SG+SD, etc.) to optimize spectral signals, achieving synergy between physical interference elimination and chemical characteristics enhancement in the core sensitive region of 4000–5500 cm^-^¹ and the 5500–7500 cm^-^¹ combined frequency region (**Figures 3**-**4**); further, using the competitive adaptive reweighted sampling (CARS) algorithm to screening feature band (Zhang et al., 2023)2. In-depth exploration of various preprocessing methods (single and combined) under different growth periods within the Collaborative optimization mechanism (e.g., MSC+FD for high-dispersion stages, SG+SD for weak-signal stages), rather than simple stacking, to achieve efficient spectral information purification (Tan et al., 2025). 3. Construction of a complete technical system of “growth period specificity-preprocessing collaboration-model adaptation” to validate the performance improvement of the Stage-based modeling strategy compared to a general model (Cao et al., 2021), providing a directly applicable solution for precision orchard management.

To this end, this study first analyzes the distribution characteristics of leaf total phosphorus (LTP) in korla fragrant pear across different growth periods using Box plot, clarifying the content dynamics during the fruit-setting period (minimum 0.02%, maximum 0.25%, left-skewed distribution), fruit-expanding period (median 0.15%, concentrated in 0.10%–0.20%), and maturity period (maximum 0.45%, right-skewed distribution) (**Figure 2**), providing a physiological basis for Spectral modeling; secondly, integrating MSC, SG smoothing, FD/SD derivative processing, and combined strategies (MSC+FD, SG+SD, etc.) to optimize spectral signals, achieving synergy between physical interference elimination and chemical characteristics enhancement in the core sensitive region of 4000–5500 cm^-^¹ and the 5500–7500 cm^-^¹ combined frequency region (**Figures 3**-**4**); further, using the competitive adaptive reweighted sampling (CARS) algorithm to screening feature band (Zhang et al., 2023), combined with algorithms such as BP neural network (Yang et al., 2021)and random forest (Capitaine et al., 2021)to construct Growth-period-specific model, with model performance evaluated and compared through metrics including Coefficient of determination (R²) and Root mean square error (RMSE).

This study not only improves the Growth period adaptation theory for Spectral diagnosis of fruit tree Phosphorus, but also provides a Methodological reference for the application of Near-infrared technology in Precise orchard nutrient management. As an important component of a series of studies, this result corroborates previous Spectral diagnosis research on plants such as potato and rice (Zhang et al., 2019; Gao et al., 2023), collectively revealing the coupling pattern of ‘spectral trait-growth period-nutritive index’, thus laying the foundation for constructing a universal technical system for fruit nutrition diagnosis (Xiao et al., 2022; Guo et al., 2024). Subsequent research will focus on the integration of Mid-infrared spectroscopy and near-infrared spectrum, as well as correction mechanisms for field environmental interferences, promoting the translation of Spectral diagnosis technology from laboratory research to practical application.

## 2 Materials and methods

### 2.1 Overview of the test site

The experiment was conducted at the campus of Tarim University in Alar City, Xinjiang. The test material was 23-year-old Korla Fragrant Pear (grafted onto Birchleaf Pear rootstock), planted in north-south rows with a spacing of 2 m×4 m. The orchard was irrigated using the Flood irrigation method, and other management practices were carried out according to local conventional protocols. Mature trees with vigorous growth and uniform tree vigor were selected for the study. Please refer to **Figure 1** for the experiment process.

### 2.2 Sample collection

At the fruit bearing periods (April 23, 2024), fruit expansion period (July 11, 2024), and maturity period (September 20, 2024) of Kuerle fragrant pear fruit, mature leaves were collected from the middle and lower segments of current-year branches at the outer edge of the tree crown of each Test tree. During collection, single leaves from the east, south, west, and north directions of the tree crown were carefully selected. Leaves from 150 trees were collected for each period, labeled, and stored in Ziplock bag inside a 4°C refrigerator for subsequent Spectral scanning and Total phosphorus content analysis.

### 2.3 Original spectrum acquisition

Remove Test sample from the -4°C freezer and place it in the laboratory where the spectrometer is located (ambient temperature 24°C) for 12 hours to equilibrate, ensuring that the sample temperature is consistent with room temperature and eliminating interference from temperature gradients (Zheng et al., 2023). After powering on the Fourier Transform Near-Infrared Spectrometer (Antaris II FT-NIR) and allowing it to warm up for 30 minutes, perform Diffuse reflectance correction using the Standard whiteboard (He et al., 2023). If necessary, gently clean dust from the leaf surface with a dust-free cloth. On the leaf, select two regions each at the upper and lower ends, using the vein as a boundary (four sites in total), and use different colors to mark the spectra of different regions. At each region, repeat the scan 4 times using the following parameters: Spectral range 10000–4000 cm^-^¹, Resolution 8 cm^-^¹, Gain 2×, Number of accumulations per scan 64 times (Zheng et al., 2024). Single leaf yielded 16 spectral curves, and after baseline correction, the average was calculated and used as the final Absorbance (A) value of the sample for subsequent Chemometric modeling and analysis. This method effectively controlled the effects of Temperature fluctuation, Instrument drift, and Leaf heterogeneity through Standardized preprocessing, Instrument calibration, and Multi-point repeated measurement, laying the data foundation for constructing a High-precision prediction model.

### 2.4 Determination of total potassium in korla fragrant pear leaves

Collect the Leaf sample after Spectral data acquisition and sequentially wash with tap water, 0.1% detergent solution, tap water, and Distilled water (entire process ≤ 2 min). After removing surface moisture with Dust-free absorbent paper, place the sample in a 105°C Forced air drying oven for fixation for 20 min, then dry to constant weight at 80°C (Dayton et al., 2017); grind the dried sample using a Stainless steel crusher and pass through a 60-mesh nylon sieve (Xu et al., 2016). Accurately weigh 0.2000 g of the Sieved sample into a 100 mL Digestion tube. Moisten the sample with Distilled water, then add 5 mL of Concentrated sulfuric acid (H_2_SO_4_), and fit the mouth of the tube with a curved neck funnel. On a Digestion furnace, initially heat gently at low temperature; gradually increase the temperature after dense white smoke appears due to decomposition of sulfuric acid. When the solution turns brown-black, remove the Digestion tube, cool, then add 10 drops of 300 g·L^-^¹ hydrogen peroxide (H_2_O_2_) dropwise while thoroughly shaking. Continue heating for 15 min. Repeat the above H_2_O_2_ addition operation 2–3 times until the Digestion solution becomes colorless or clear and transparent, then heat for an additional 10 min to completely remove excess H_2_O_2_ (Zou et al., 2019); after cooling, rinse the curved neck funnel with Distilled water, combine the rinsing solution into the Digestion tube, and dilute to the 100 mL mark; determination of Total phosphorus content is performed using the molybdenum antimony resistance colorimetric method (Li Ting and Hong Xing, 2022), specifically: transfer 5 mL of Digestion solution (pre-dilute if concentration is too high) into a 50 mL volumetric flask, add 5 mL of freshly prepared molybdenum-antimony-ascorbic acid color developer [preparation: slowly add 100 mL of 0.5 mol/L H_2_SO_4_ to a mixed solution containing 10 g ammonium molybdate and 0.5 g antimony potassium tartrate, cool, then add 1.5 g of Ascorbic acid and dilute to 500 mL], dilute to volume with Distilled water, and allow color development in the dark at 20–30°C for 30 min; using the Blank solution as reference, measure the absorbance at 700 nm, and calculate the Total phosphorus content of the sample using Equation 1:


(1) $$LTP\left(\%\right)=\frac{C\times V\times D}{m\times 10^{4}}$$

where C is the measured concentration (mg/L), V is the final volume (100 mL), D is the dilution factor, and m is the sample weight (0.2000 g)

### 2.5 Spectrum data conversion

In the spectral data processing process, specific Spectral transformation can be used to mitigate the effects of environmental factors and interferences, improve the Signal-to-noise ratio, and make the spectral form more suitable for Korla fragrant pear LTP. In this study, several mathematical transformations were applied to the original spectra, generating six types of spectral data: Original absorbance (A), MSC, SG, FD, SD, MSC+FD,MSC+SD, SG+FD, SG+SD (As shown in **Table 1**). MSC is a Normalization technique that reduces baseline drift, improves the Signal-to-noise ratio, and better reveals differences and similarities among samples; it is commonly used to eliminate scattering effects on spectral data (Gautam et al., 2015). SG is a Local smoothing method based on Polynomial fitting, which performs Weighted filtering on Spectral data through a Sliding window, achieving Random noise reduction while preserving Peak shape and Spectral details (Liu et al., 2016). FD can enhance the Resolution of spectra, distinguish Overlapping peaks, and is suitable for determining Peak position and boundary, especially for eliminating Background interference in Quantitative analysis (Sonobe and Hirono, 2023). SD is more sensitive in identifying Peak inflection points and shoulder peaks, and suppresses the Broad background signal, commonly used in the analysis of fine structures in complex spectra (Gao et al., 2016). MSC+FD first corrects the Scattering effect using MSC, then applies First-order derivative (FD) to eliminate residual baseline drift and improve peak Resolution, making it suitable for scenarios with strong scattering interference and requiring precise Peak positioning (Wang et al., 2023). MSC+SD enhances spectral details after MSC reduces scattering, with the second-order derivative (SD) further identifying subtle differences in overlapping peaks, suitable for complex samples requiring resolution of highly overlapping peaks (Xie et al., 2018). SG+FD first applies SG smoothing to reduce noise, then calculates the first-order derivative (FD), avoiding amplification of noise in derivative results, thus balancing noise suppression and resolution enhancement in spectra with high noise levels (Zhou et al., 2024). SG+SD first uses SG smoothing to reduce noise, after which the second-order derivative (SD) more accurately reflects spectral curvature, avoiding false peaks caused by noise, making it suitable for spectra requiring fine structure analysis where noise is significant (An et al., 2021).

**Table 1:** Spectral preprocessing methods.

Full name	Abbreviation	Function
Multiple Scattering Correction	MSC	Eliminate scattering
Savitzky-Golay smoothing	SG	Reduce noise
First derivative	FD	Enhance spectral resolution
Second derivative	SD	Conduct fine structural analysis
Multiple Scattering Correction+ First Derivative	MSC+FD	Eliminate scattering + Enhance spectral resolution
Multiple Scattering Correction+Second Derivative	MSC+SD	Eliminate scattering + Conduct fine structural analysis
Savitzky-Golay+First Derivative	SG+FD	Reduce noise + Enhance spectral resolution
Savitzky-Golay+Second Derivative	SG+SD	Reduce noise + Conduct fine structural analysis

### 2.6 Extraction of spectrum characteristic bands

To reduce the band redundancy and interference of high-dimensional spectral data, feature bands significantly associated with leaf total phosphorus content (LTP) (LTP) were selected from the spectral data to improve modeling accuracy. In this study, the Competitive adaptive reweighted sampling (CARS) algorithm (Sun et al., 2021; Xing et al., 2021) was adopted, based on the principle of darwin’s theory of biological evolution’s survival of the fittest. Efficient dimensionality reduction of spectral variables was achieved by coupling Partial least squares (PLS) modeling with an adaptive variable screening mechanism. This algorithm evaluates the importance of variables based on the absolute percentage evaluation of PLS model coefficients, generates an initial wavelength subset through Monte Carlo sampling (MCS), and dynamically adjusts variable weight by incorporating an Exponential decay function to strengthen the selective retention of high-contribution bands. Simultaneously employing the Adaptive weighted sampling (ARS) strategy, wavelengths are weighted screening based on the Absolute value of coefficients, prioritizing the retention of Important bands and eliminating Redundant information. After Multi-round iterative optimization, the final Characteristic wavelength combination highly correlated with LTP is obtained, providing an efficient Input variable set for subsequent Regression modeling.

### 2.7 Machine learning modeling

Based on the above trait selection results, three algorithms—Random forest (RF), Support vector machine (SVR), and Back Propagation(BP)neural network—were used to construct a Korla Fragrant Pear LTP estimation model.

RF (Guo and Hao, 2021)reduces model variance by integrating multiple decision trees, with core parameter settings as follows: Number of decision trees (n _ estimators) was set to four gradients—100, 250, 500, and 750—where the smaller value (100) was used to explore the model baseline performance, medium values (250, 500) balanced computational efficiency and ensemble effect, and the larger value (750) verified the fitting ability under extreme ensemble scale (Rhodes et al., 2023); min_samples_leaf (min_samples_leaf) was set to five gradients—1, 3, 5, 7, and 10—where the minimum value (1) allowed the decision tree to grow sufficiently to capture subtle variations, medium values (3, 5) suppressed noise dissociation, and larger values (7, 10) enforced simplification of tree structure to reduce complexity (Jeong et al., 2016; Scornet, 2016).

SVR (Virnodkar et al., 2020)employed four types of kernel functions for comparison: Radial basis kernel function (RBF), Linear kernel function, Polynomial kernel function, and Sigmoid kernel function. The optimal parameter values of the penalty coefficient (C) and the kernel function parameter (γ) were determined through grid search optimization, thereby identifying the optimum parameter and the kernel function.

BP neural network (Li et al., 2021)consists of input, output, and an intermediate hidden layer. A single hidden layer is used, with the number of nodes set to 1–10. The model iterates 1000 times, with a learning rate of 0.01 and a training target error of 1×10^-^⁶. Six training functions (trainlm, traingd, trainscg, traingdx, trainbfg, and traincgb) are compared to determine the optimal parameters and the best training function (Wang et al., 2023).

This paper conducts comprehensive comparative experiments with the currently recognized advanced baseline model in the field. For this, we selected three high-performing and widely used Representative model for spectral analysis as advanced representatives of the baseline model. Partial least squares regression (PLSR) and Light gradient boosting machine (LightGBM) and One-dimensional convolutional neural network (1D-CNN).

PLSR is a Regression modeling method proposed in the early 1980s by Svante Wold and others for handling High-dimensional, multicollinear data (Li et al., 2024). It achieves prediction of Y by extracting Latent Variables (Latent Variables) from the Independent variable (X) and Dependent variable (Y), and establishing a linear regression relationship between these latent variables. It is particularly suitable for the Chemometrics field such as Spectroscopy and Chromatography. LightGBM was proposed in 2017 by Microsoft Research Asia, and is an efficient implementation of the Gradient Boosting Decision Tree (GBDT) framework, greatly improving Training speed and reducing Memory consumption, while maintaining high prediction accuracy, making it perform exceptionally well on Large-scale data (Gupta et al., 2021).1D-CNN originated from the LeNet-5 architecture proposed by Yann LeCun and others in the late 1980s and early 1990s for Handwritten digit recognition (designed for 2D images), which was the prototype of the Convolutional Neural Network (CNN). 1D-CNN can automatically learn Local patterns and Multi-scale features in data, making it highly suitable for processing one-dimensional signal such as time series analysis, audio frequency processing, and Near-infrared spectroscopy (Liu et al., 2022).

### 2.8 Medel evaluation methods

This study implemented the aforementioned Regression algorithm using MATLAB R2024b, and comprehensively evaluated Model performance using three metrics: Coefficient of determination (R²), Root mean square error (RMSE), and Residual prediction deviation (RPD):

R²: Measures Model goodness of fit. The value ranges from 0 to 1. The closer R² is to 1, the higher the agreement between the Model predicted value and the measured value (Ahmad Yasmin et al., 2021). The calculation formula is shown in Equation 2.

RMSE: Quantifies the absolute magnitude of Prediction error. The smaller the RMSE value, the higher the Model prediction accuracy (Li et al., 2024). The calculation formula is shown in Equation 3.

RPD: Reflects the predictive capability of the model. Evaluation criteria: RPD > 3: Excellent Model prediction ability; 2< RPD ≤ 3: The model can be used for preliminary prediction; RPD ≤ 2: Poor Model prediction ability. The calculation formula is shown in Equation 4.In model evaluation, the Dataset is divided into training sets and Test set at a ratio of 3:1, and the above metrics are calculated separately to comprehensively assess the model’s Fitting effect, Prediction accuracy, and Generalization ability (Chu et al., 2023).


(2) $$R^{2}=1-\frac{\sum_{i=1}^{n}{\left(y_{m}-y_{p}\right)}^{2}}{\sum_{i=1}^{n}{\left(y_{m}-\bar{y}\right)}^{2}}$$

(3) $$RMSE=\sqrt{\frac{1}{n}\sum_{i=1}^{n}{\left(y_{m}-y_{p}\right)}^{2}}$$

(4) $$RPD=\frac{S_{y}}{RMSE}=\frac{\frac{1}{n-1}\sum_{i=1}^{n}{\left(y_{m,i}-{\bar{y}}_{m}\right)}^{2}}{PMSE}$$

the sample size; ${\text{y}}_{\text{m}}$ and ${\text{y}}_{\text{p}}$ They are the actual value and the predicted value of Korla fragrant pear Leaf Total Potassium respectively; $\bar{y}$ It is the average value of the actual Korla fragrant pear Leaf Total Potassium; ${\text{S}}_{y}$ Is the standard deviation of the Leaf Total Potassium measurement value of Korla fragrant pear

## 3 Results and analysis

### 3.1 Content of korla fragrant pear total phosphorus in leaves at different periods

As shown in **Figure 2** Box plot, the LTP content in Korla Fragrant Pear leaves exhibited significant distribution differences across various growing stage, providing a basis for Stage-based modeling. Samples from the fruit-setting period generally had lower overall content but included individual high-value outliers, reflecting periodic fluctuations in Phosphorus demand during this stage. This distribution facilitates the model’s ability to capture differences in Spectral response under low and high phosphorus conditions. The distribution of LTP content during the fruit swelling period was relatively concentrated with low dispersion, indicating more stable Phosphorus levels at this stage, allowing modeling to focus on identifying representative Spectral characteristics. The maturity period showed more high-value samples and extreme high values, which, while increasing the difficulty of model identification, also provided critical sample support for establishing Quantitative prediction within the high-content range. The above distribution characteristics indicate that there are significant differences in the Growth period LTP content and their degree of variation among various periods. Therefore, adopting a unified prediction model is unlikely to achieve global optimization. Instead, it is necessary to construct specificity model based on the data characteristics of each period to improve Prediction accuracy and robustness.

### 3.2 Analysis of leaf spectral data in korla fragrant pear

This study is based on the use of Near-Infrared Spectroscopy (4000–10000 cm^-^¹) to analyze the Leaf total phosphorus (LTP) content of Korla fragrant pear. Differences in leaf LTP content exist during Different growth stages, manifested as Fruit-setting period< Fruit expansion period< Maturity period, providing a sample basis for Stage-based modeling. It provides a sample foundation for Spectral modeling.

Spectral response indicates that changes in LTP content are significantly associated with the Vibrational absorption of Hydrogen-containing group (such as P-O-H). In the 4000–5500 cm^-^¹ range, the Hydrogen group overtone absorption of Phosphorus substance overlaps with that of moisture and Carbohydrates, forming the Core sensitive region for LTP; the Combination frequency and overtone in the 5500–7500 cm^-^¹ range can synergistically and complementarily verify differences in total phosphorus (**Figure 3A**).The line discretization and polymerization of Spectrum curves from different samples reflect the group differences in LTP content. Combined with the Growth period classification of Spectrum plots, distinct dispersion and polymerization of curves in characteristic intervals across different periods are observed (**Figure 3B**), further providing a basis for establishing the Stage-based prediction model.

### 3.3 Spectral data preprocessing

This study applied MSC, SG, FD, SD and their combined methods to preprocess the near-infrared spectra of Korla fragrant pear leaves, aiming to enhance the Spectral characteristics associated with LTP content while reducing noise and scattering interference.

The Original spectrum was influenced by baseline effects and noise, leading to significant signal overlap and indistinct variations (**Figure 3A**). MSC effectively removed baseline drift caused by particle size and surface scattering, markedly improving spectral consistency and comparability (**Figure 4A**); SG suppressed random noise while preserving the original Peak shape, thereby increasing the Signal-to-noise ratio and help to highlight phosphorus-related absorption features (**Figure 4B**); FD processing amplified the dynamic differences of absorption peak related to LTP content by calculating the spectral rate of change, which improved feature discernibility (**Figure 4C**); SD further accentuated subtle changes in Spectral curvature, proving particularly useful for extracting weak signals from low-content samples (**Figure 4D**).Combined preprocessing strategy integrates the advantages of individual methods, further enhancing Spectrum quality. MSC+FD eliminates physical scattering while amplifying dynamic spectral features, making it more effective at capturing variations in LTP content (**Figure 4E**); MSC+SD improves detail resolution on the basis of scatter correction, supplying the model with more stable and refined input (**Figure 4F**); SG+FD preserves and accentuates spectral changes induced by chemical constituents while reducing noise, thereby balancing the need for Smooth and feature enhancement (**Figure 4G**); SG+SD achieves both noise suppression and high-frequency detail enhancement, making it suitable for dynamic monitoring and Modeling of LTP content across the whole growth period (**Figure 4H**).

The results demonstrate that different preprocessing methods improve Spectrum quality in various aspects, including noise suppression, removal of physical interference, and enhancement of dynamic features. Combined methods exhibit stronger adaptability and synergistic Gain effects. Subsequently, the optimal pretreatment strategy will be selected based on Model performance metrics, laying the groundwork for high-accuracy prediction of LTP content.

### 3.4 Spectral data and correlation analysis with LTP

**Figure 5:**
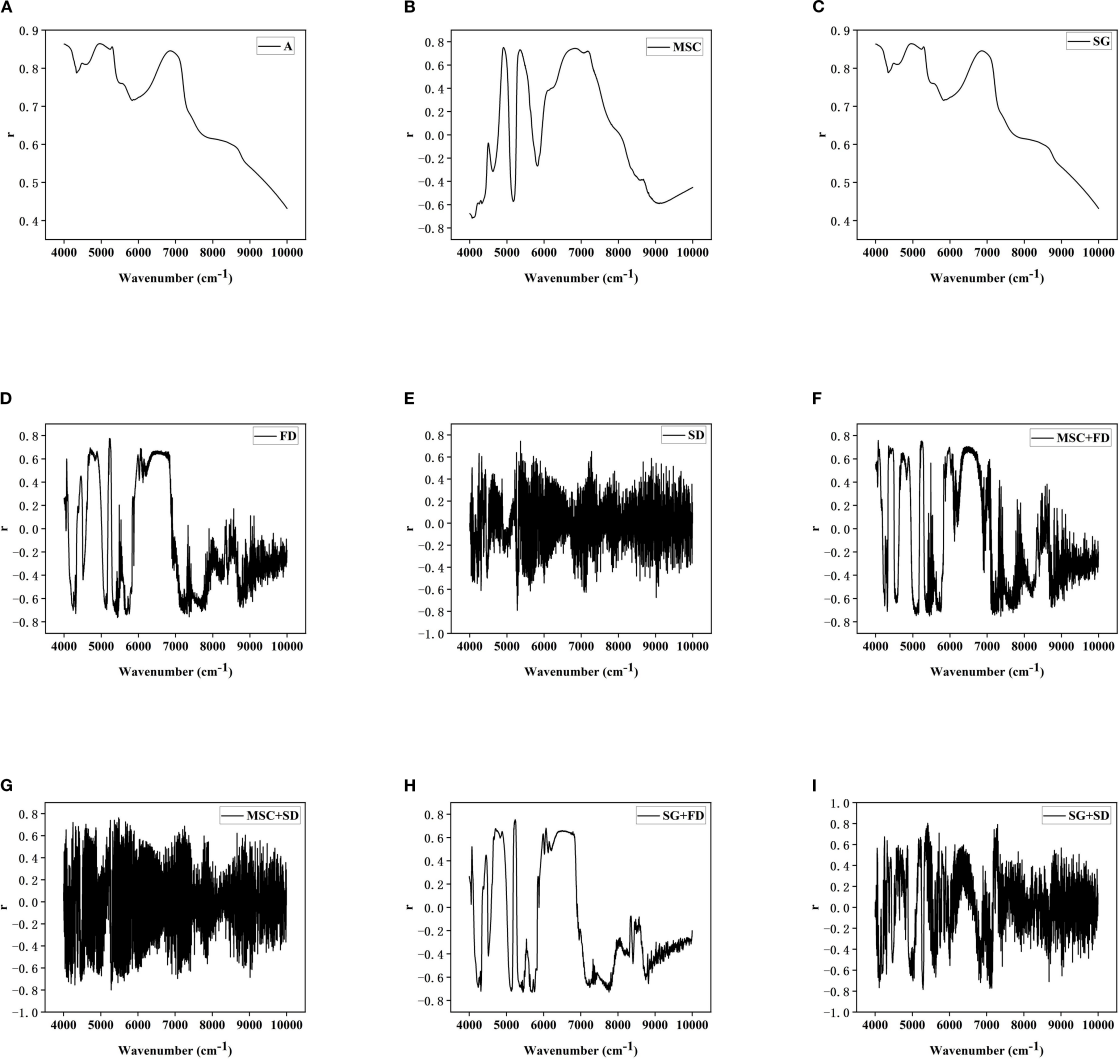
**(A)** Original spectrum; **(B)** MSC; **(C)** SG; **(D)** FD; **(E)** SD; **(F)** MSC+FD; **(G)** MSC+SD; **(H)** SG+FD; **(I)** SG+SD.

This study evaluated the optimization effects of various methods on Phosphorus information extraction by analyzing the Correlation (r) between different Preprocessed spectra and LTP content. The main findings are summarized below (representative r values are provided in **Figure 5**):

The Original spectrum (**Figure 5A**) displays broad and smooth Peak shape, rendering it susceptible to scattering and Noise interference; Multiplicative scatter correction processing (**Figure 5B**) effectively suppressed physical interference; Savitzky-Golay processing (**Figure 5C**) showed no noticeable improvement; First Derivative processing (**Figure 5D**) amplified both Dynamic correlation details and Noise interference; Second Derivative processing (**Figure 5E**) improved the ability to extract trace phosphorus-containing components and detect Weak correlation with LTP.

Combined preprocessing methods exhibited stronger Synergistic effect. The MSC+FD approach (**Figure 5F**) significantly enhanced the Recognition ability of LTP in Highly discrete samples; MSC+ SD (**Figure 5G**) improved the resolution of LTP information in Low phosphorus content samples; SG+ FD(**Figure 5H**) strengthened the correlation between spectral data and LTP while enhancing Interpretability; SG+ SD (**Figure 5I**) reduced Noise interference and accentuated Weak absorption difference, thereby improving the Dynamic monitoring ability of LTP during Different growth stages. In summary, the Combined pre-processing method can more effectively extract Spectral characteristics associated with LTP content, thereby providing a more reliable data foundation for subsequent Modeling.

Correlation Analysis between Different Spectral Data and LTP in **Figure 5**: (A) Original spectrum; (B) MSC; (C) SG; (D) FD; (E) SD; (F) MSC+FD; (G) MSC+SD; (H) SG+FD; (I) SG+SD.

### 3.5 Selection of LTP characteristic bands in korla fragrant pear

**Figure 6:**
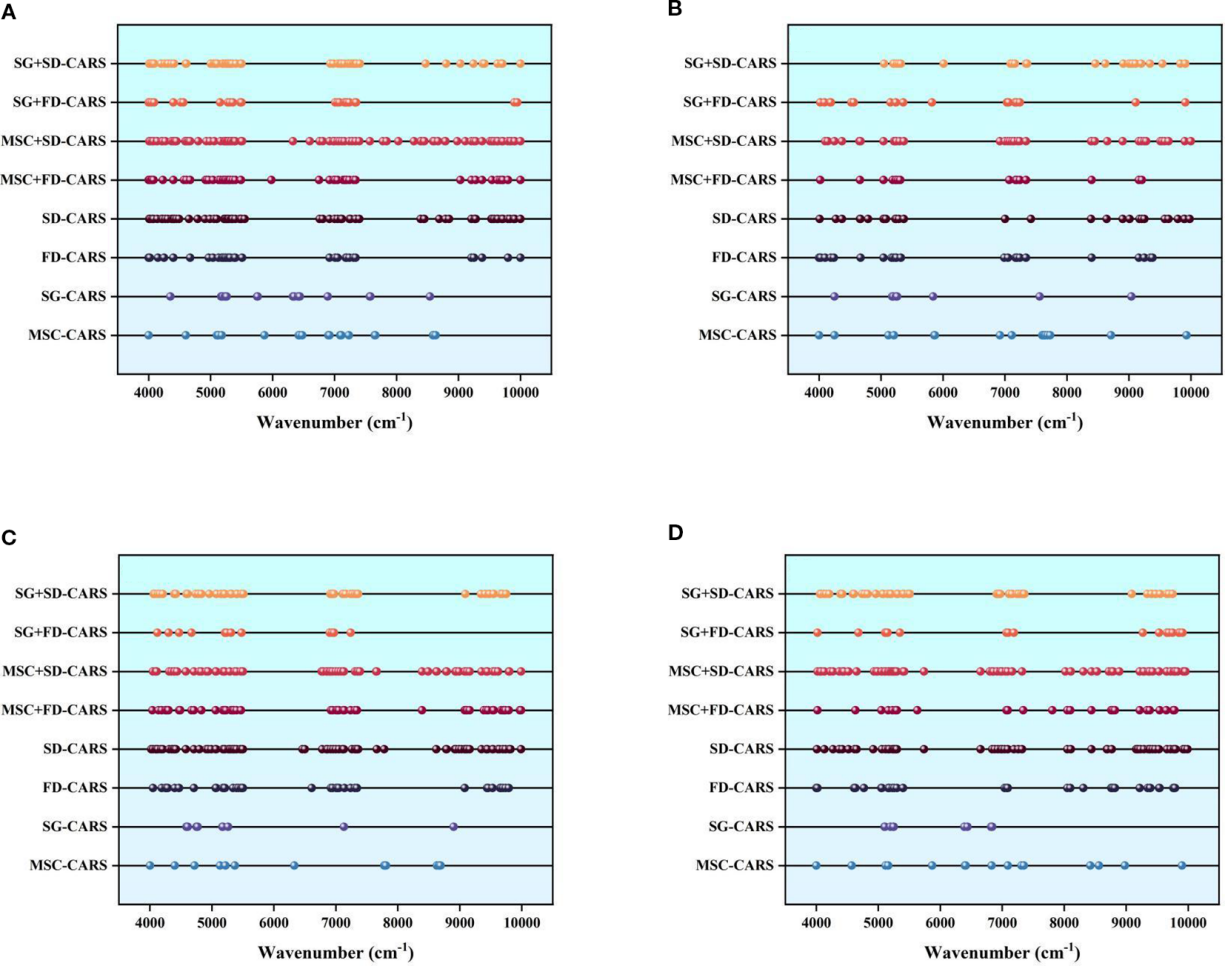
Selection of korla fragrant pear LTP characteristic bands: **(A)** represents the selection for the spectral data of the whole growth period; **(B)** represents the selection for the spectral data of the fruit bearing periods; **(C)** represents the selection for the spectral data of the fruit swelling period; **(D)** represents the selection for the spectral data of the fruit ripening period.

This study utilized the Competitive Adaptive Reweighted Sampling algorithm to screening feature bands highly correlated with LTP content from preprocessed near-infrared spectra, and analyzed the distribution characteristics of these bands across both the whole growth period and Different growth stages (as shown in **Figure 6**).

The results demonstrate that combined preprocessing methods (such as MSC+FD,SG+SD) consistently extracted denser and more comprehensive feature bands across all Different growth stages, proving particularly effective at capturing subtle chemical absorption variations. In contrast, single preprocessing methods (e.g., Multiplicative scatter correction or Second Derivative) mainly focused on core absorption peak regions, resulting in fewer but more specific feature bands.

Regarding growth stage differences: during the fruit-setting stage, LTP content was high and exhibited high variability. Combined preprocessing methods produced densely clustered feature bands in certain spectral regions, effectively adapting to high-phosphorus absorption variations and capturing dynamic differential features. During the fruit-expansion stage, LTP content remained stable, and feature bands were more uniformly distributed across the 4000–8000 cm^-^¹ range. Combined methods formed continuous characteristic zones in some bands, aligning with chemical equilibrium states during this stable period and enabling the identification of more comprehensively correlated bands. In the maturity stage, LTP content was low and absorption signals were weak, making it necessary to rely on combined preprocessing to enhance the extraction of bands sensitive to trace components. The Feature band selection results provide critical input for building subsequent staged LTP content prediction models. By leveraging Growth period characteristics, suitable Pretreatment strategies can be selected to improve model accuracy and specificity.

### 3.6 Korla fragrant pear LTP estimation model

This study constructed Growth-period-specific models and an intertemporal general model based on the LTP content and Spectral data of Korla fragrant pear leaves at Different growth stages, using Coefficient of determination (R²), Root mean square error (RMSE), and Residual prediction deviation (RPD) as evaluation metrics for Model performance (Supplementary Material 1). The results indicated that each Growth-period-specific model significantly outperformed the intertemporal general model in predicting LTP content.

As shown in **Tables 1** and **2**, the optimal model for the fruit-setting period (FD+CARS-BP) achieved R² = 0.89, RMSE = 0.0212, RPD = 3.1696 on the Training set and R² = 0.88, RMSE = 0.0241, RPD = 2.6963 on the validation set, demonstrating a stronger ability to capture the dynamic Spectral characteristics of highly discrete LTP content. Its performance was significantly superior to that of the MSC-CARS-BP general model. The optimal model for the Fruit expansion period (SG+FD-CARS-BP) achieved Coefficient of determination (R²) values of 0.86 and 0.83 on the Training set and validation set, respectively, with Root mean square error (RMSE) values of 0.0211 and 0.0254, and RPD values of 2.6721 and 2.4571, demonstrating better adaptation to the relatively stable Spectrum-chemical state during this period. The optimal model for the Maturity period (SG+SD-CARS-BP) achieved Coefficient of determination (R²) values above 0.85 on both training and validation sets, with Root mean square error (RMSE) below 0.021 and RPD exceeding 2.68, indicating effective resolution of weak Spectrum signals from low-content LTP and significantly outperforming the general model.

**Table 2:** Indicators of the best models under each machine learning algorithm in each period.

Period	Model	Training set			Validation set		
		R^2^	RMSE	RPD	R^2^	RMSE	RPD
Entire growth period	MSC+SD-CARS-RF	0.80	0.0269	2.2094	0.76	0.0334	2.0521
	MSC-CARS-BP	0.84	0.0258	2.4436	0.81	0.0268	2.3764
	MSC+FD-CARS-SVM	0.79	0.0287	2.1862	0.78	0.0290	2.0917
Fruit setting period	SG+FD-CARS-RF	0.85	0.0267	2.5807	0.77	0.0295	2.0965
	FD+CARS-BP	0.89	0.0212	3.1696	0.88	0.0241	2.9663
	MSC+SD-CARS-SVM	0.84	0.0279	2.2024	0.79	0.0281	1.9787
Fruit Enlargement Stage	MSC+SD-CARS-RF	0.80	0.0257	2.2293	0.82	0.0238	2.3391
	SG+FD-CARS-BP	0.86	0.0211	2.6721	0.83	0.0254	2.4571
	MSC-CARS-SVM	0.90	0.0194	4.3079	0.81	0.0228	2.2185
Fruit Ripening Stage	SG+FD-CARS-RF	0.81	0.0231	2.3696	0.80	0.0237	2.2457
	SG+SD-CARS-BP	0.86	0.0203	2.6977	0.85	0.0207	2.6844
	MSC-CARS-SVM	0.82	0.0227	3.0751	0.86	0.0218	2.2088

**Figure 7:**
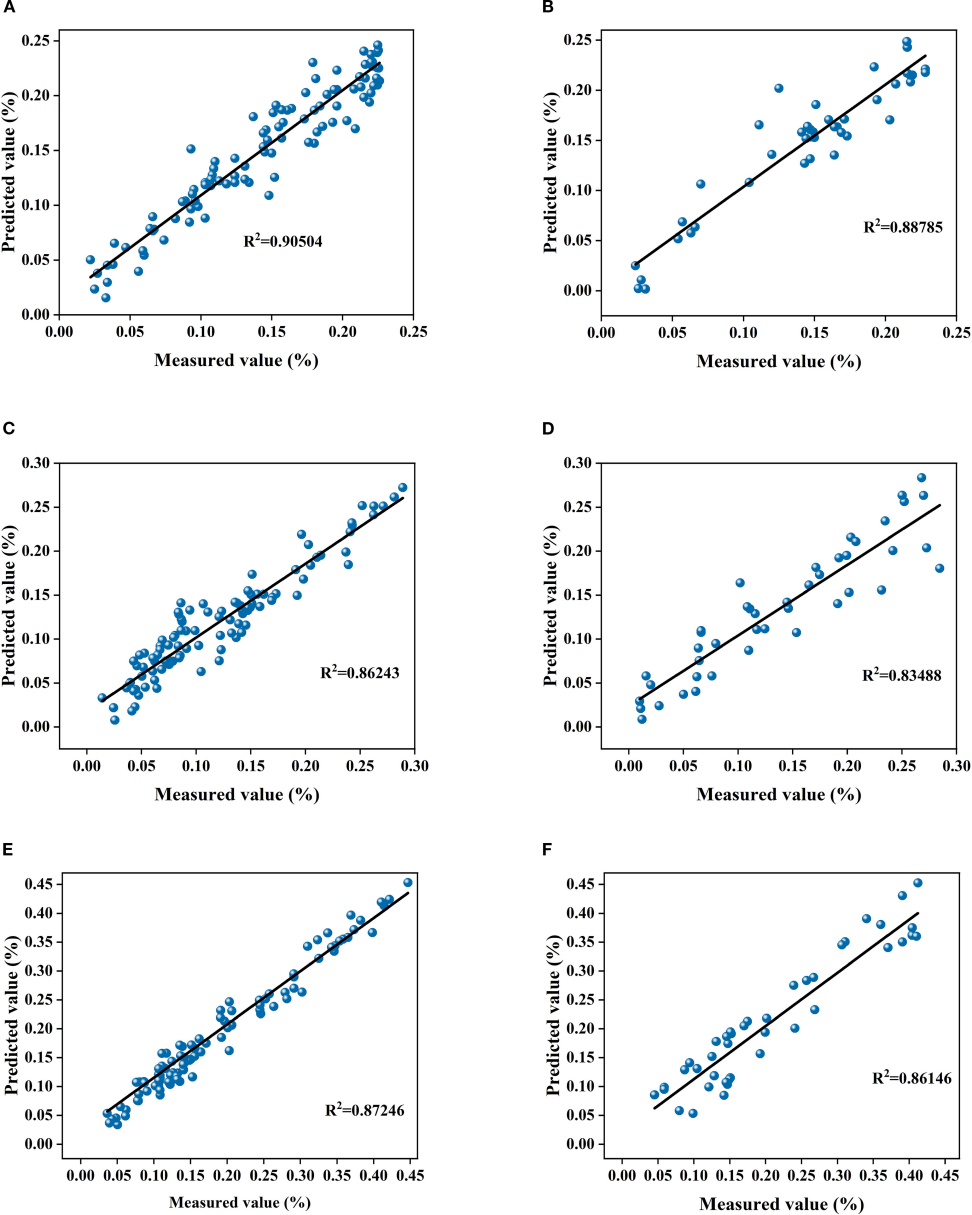
**(A)** shows the linear fit between the measured and predicted values of the training sets for the fruit bearing periodsFD+CARS-BP model; **(B)** shows the linear fit between the measured and predicted values of the validation set for the fruit bearing periodsFD+CARS-BP model; **(C)** shows the linear fit between the measured and predicted values of the training sets for the fruit swelling periodSG+FD-CARS-BP model; **(D)** shows the linear fit between the measured and predicted values of the validation set for the fruit swelling periodSG+FD-CARS-BP model; **(E)** shows the linear fit between the measured and predicted values of the training sets for the fruit ripening periodSG+SD-CARS-BP model; **(F)** shows the linear fit between the measured and predicted values of the validation set for the fruit ripening periodSG+SD-CARS-BP model.

Linear fitting results (**Figure 7**) further support the above conclusions. The Coefficient of determination (R²) values for the Fruit-setting period model reached 0.905 and 0.888 on the training and validation sets, respectively, indicating its strong fitting and Generalization ability even with highly Variant data. The Coefficient of determination (R²) values for the Fruit expansion period model were all above 0.83, and those for the Maturity period model exceeded 0.86, further confirming the adaptability and stability of the specificity model across Different growth stages. Studies have shown that the Growth-period-specific model, by aligning with the differences in LTP content and Spectral characteristics across various periods, significantly improves prediction accuracy, providing a reliable model option for precise phosphorus nutrition management in orchards. Therefore, it is recommended to use the FD+CARS-BP model during the fruit-setting period, the SG+FD-CARS-BP model during the fruit swelling period, and the SG+SD-CARS-BP model during the maturity period for predicting the Korla fragrant pear LTP content.

### 3.7 Model parameters and function selection

**Figure 8:**
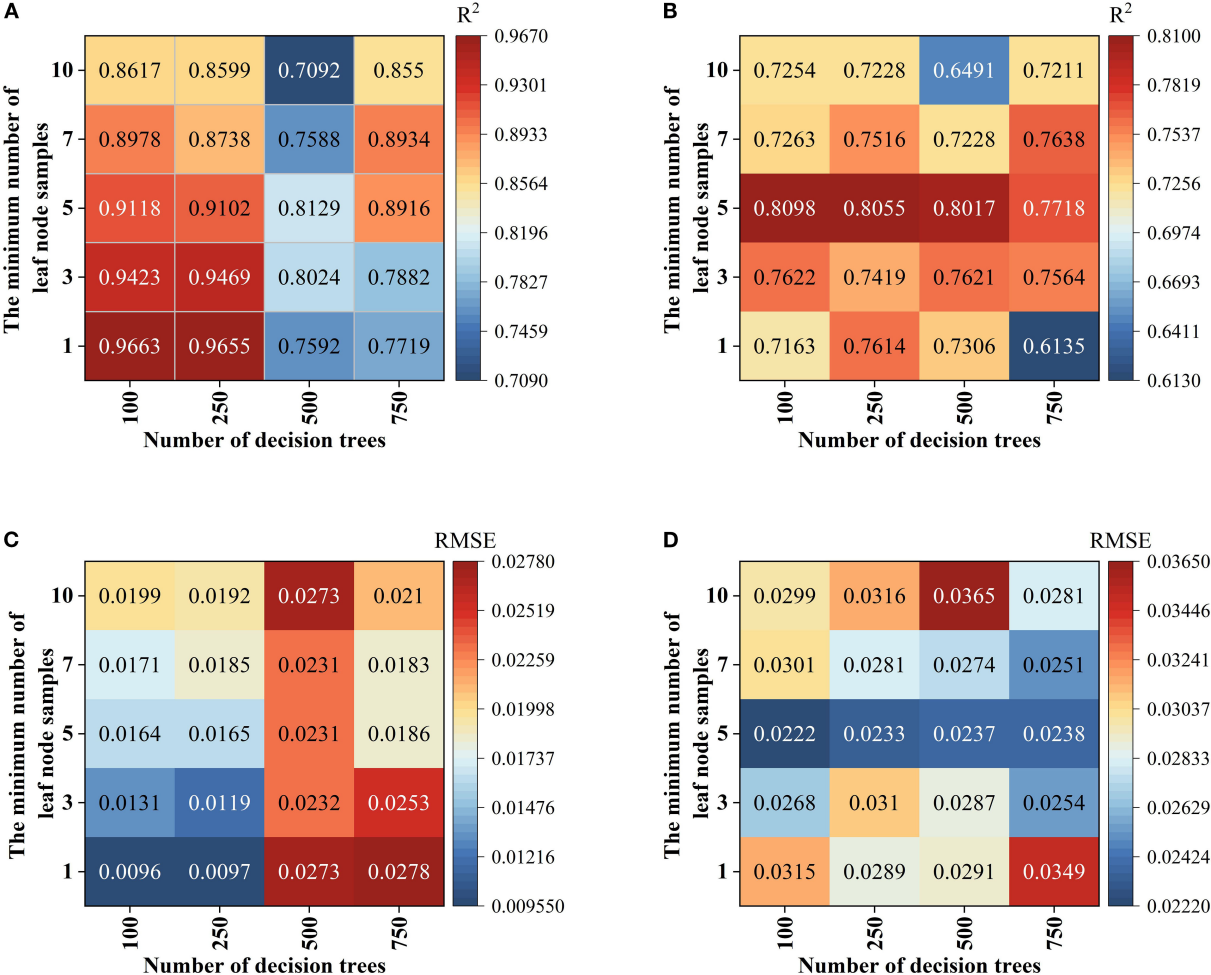
RFhyper parameter settings: **(A)** is R2 of training sets; **(B)** is Root mean square error (RMSE) of training sets; **(C)** is R2 of the validation set; **(D)** is Root mean square error (RMSE) of the validation set.

**Figure 9:**
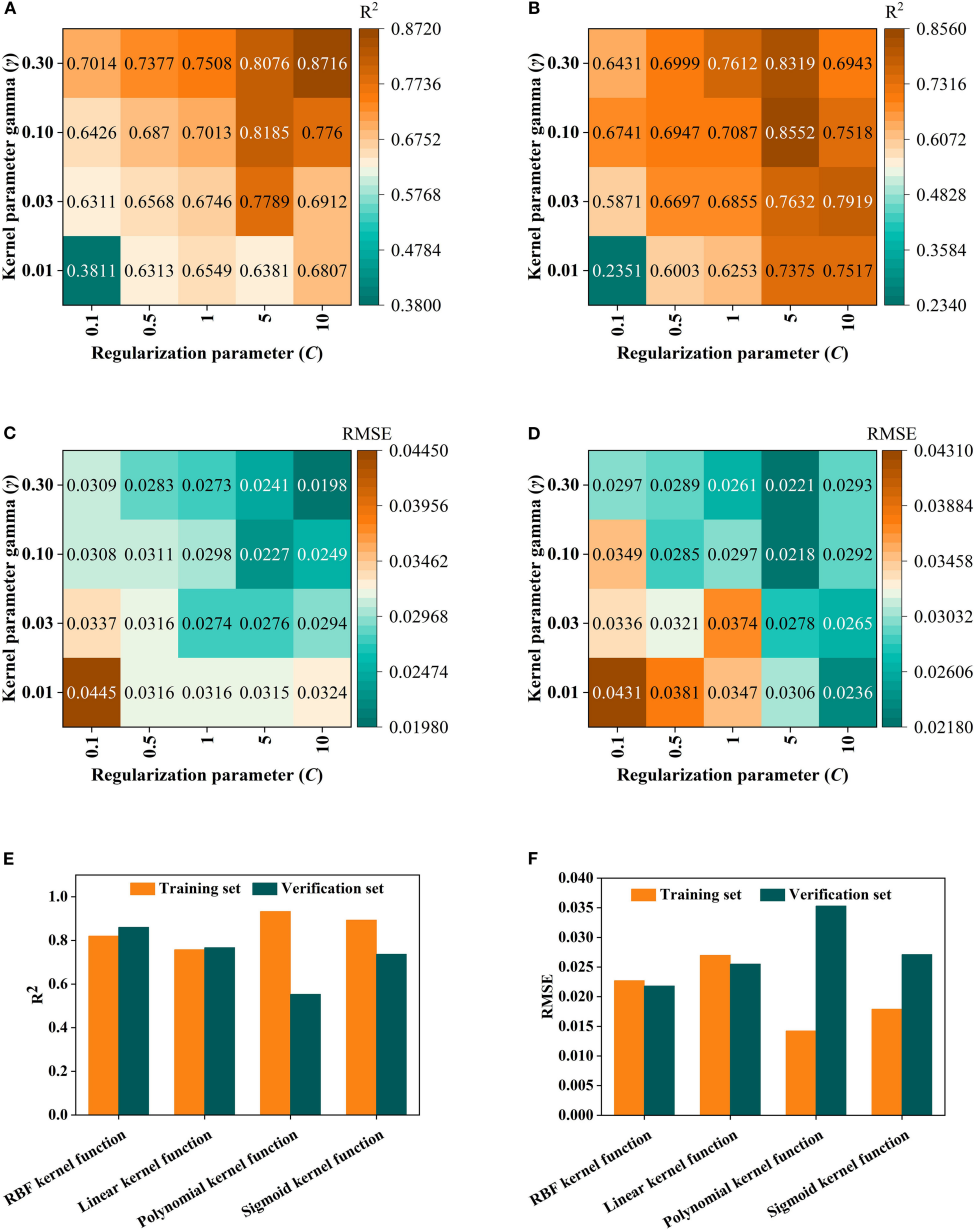
SVM hyper parameter settings and kernel function selection: **(A)** is R2 of training sets; **(B)** is Root mean square error (RMSE) of training sets; **(C)** is R2 of validation set; **(D)** is Root mean square error (RMSE) of validation set; **(E)** is R2 of four kernel functions for training sets and validation set; **(F)** is Root mean square error (RMSE) of four kernel functions for training sets and validation set.

**Figure 10:**
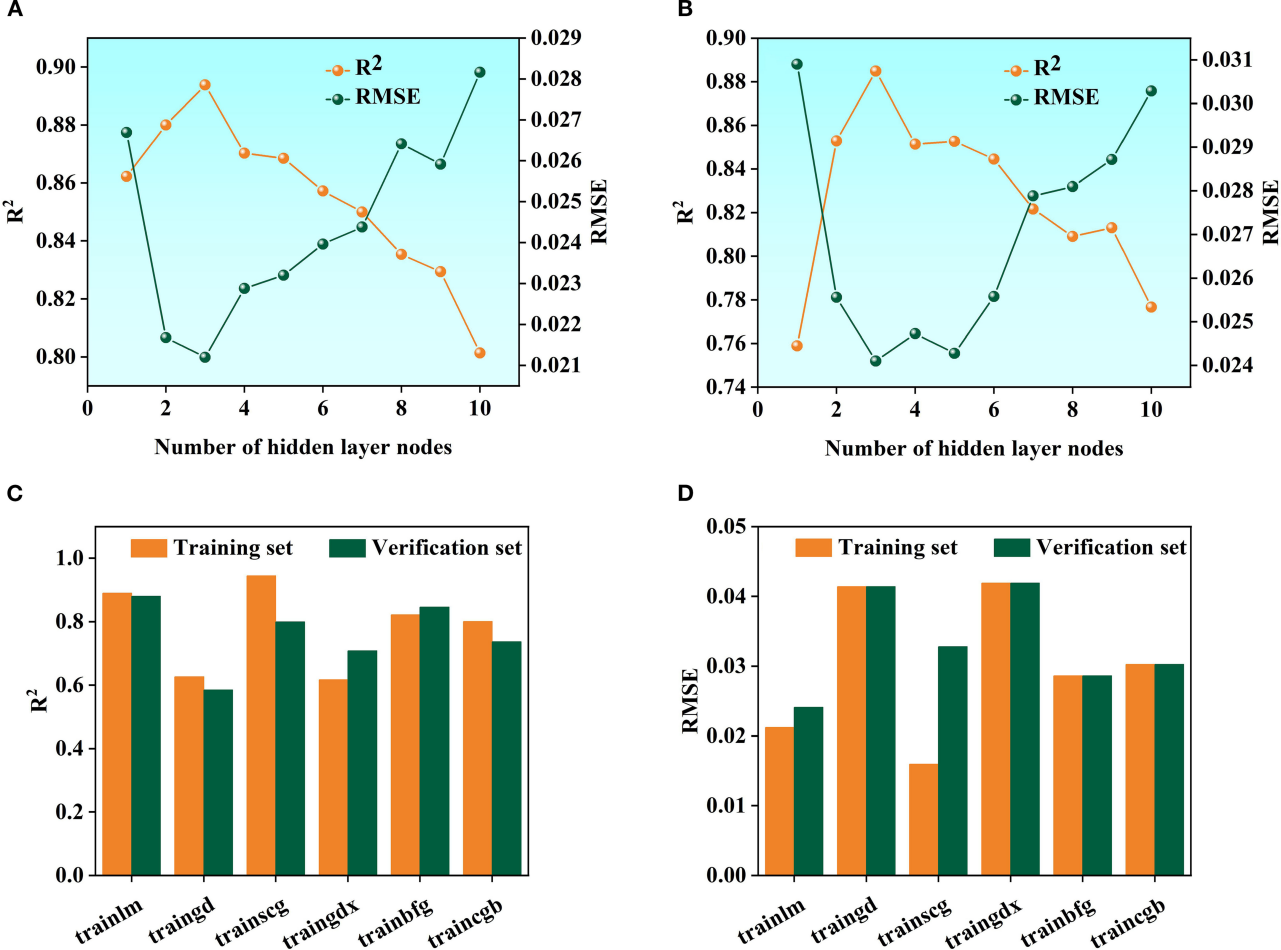
BP neural networkhyper parameter settings and kernel function selection: **(A)** is R2 and Root mean square error (RMSE) of training sets; **(B)** is R2 and Root mean square error (RMSE) of the validation set; **(C)** is R2 of six training functions; **(D)** is Root mean square error (RMSE) of six training functions.

In the Random forest (RF) Modeling process, the number of Decision trees and the value of min_samples_leaf are key hyper parameters that directly affect model complexity and Generalization ability. With too few trees, the model’s fitting ability is inadequate, leading to poor fitting. As the number of trees increases, the model improves prediction stability through ensemble learning. However, beyond a certain point, the performance Gain becomes marginal, while computational cost and Overfitting risk rise (Huang et al., 2016; Guo et al., 2019; Dabiri et al., 2022). Taking the fruit ripening period SG+FD-CARS-RF model as an example (**Figures 8A–D**), when the number of trees is 500 and min_samples_leaf is 5, the difference in Coefficient of determination (R²) between the Training set and the validation set is the smallest (0.0112), and the Root mean square error (RMSE) difference is only 0.006—significantly better than other parameter combinations. This indicates that this configuration maintains strong Generalization ability while mitigating overfitting, and was therefore identified as the optimal parameter set. For the SVM model, using the mature stage Multiplicative scatter correction-Competitive Adaptive Reweighted Sampling-SVM as an example, it is essential to optimize the regularization parameter C and the kernel parameter γ. As illustrated in **Figures 9A–D**, when C = 5 and γ=0.1, the model performs well on both the Training set and the validation set (with Coefficient of determination (R²) values of 0.8185 and 0.8552, and Root mean square error (RMSE) values of 0.0227 and 0.0218, respectively), demonstrating a good balance. Although a higher Training set Coefficient of determination (R²) of 0.8716 is achieved when C = 10 and γ=0.3, the validation set Coefficient of determination (R²) drops significantly to 0.6943, indicating clear overfitting. Thus, C = 5 and γ=0.1 are identified as the optimal parameters. Further comparison among different kernel functions shows that the Radial basis kernel function (RBF) delivers the best overall performance, with the smallest discrepancy in Coefficient of determination (R²) between the validation set and the Training set. In contrast, although the Polynomial kernel function performs well on the Training set, its validation set Coefficient of determination (R²) decreases by up to 0.3792, reflecting inadequate Generalization ability. This suggests that the RBF kernel is more suitable for the characteristics of the present dataset. In the BP neural network model, using the fruit-setting period FD+CARS-BP as an example, the number of nodes in the hidden layer significantly influences the model’s expressive power. As shown in **Figures 10A, B**, when the hidden layer contains 5 nodes, both the Training set and validation set show high Coefficient of determination (R²) and low Root mean square error (RMSE), indicating that this configuration maintains strong fitting ability without noticeable overfitting. Further comparison among different training functions shows that the trainlm function performs best in this model, achieving a validation set Coefficient of determination (R²) of 0.88 and an Root mean square error (RMSE) of 0.0241, surpassing other training functions and demonstrating its superior suitability for the given data structure and task complexity.

### 3.8 Performance comparison with advanced baseline model

To evaluate the performance of the optimal models selected for each Growth period, this study conducted a comprehensive comparison with widely recognized advanced baseline models in the field, namely PLSR, 1D-CNN, and LightGBM. Each baseline model employed the same Pretreatment and Feature band selection methods as the corresponding optimal model for the respective growth stage (Fruit-setting period: First Derivative-Competitive Adaptive Reweighted Sampling; Fruit-expanding period:SG+FD-CARS; Maturity period:SG+SD-CARS) to ensure a fair comparison (**Table 3**).

**Table 3:** Performance comparison with advanced baseline model.

Period	Model	Training set			Validation set		
		R²	RMSE	RPD	R²	RMSE	RPD
Fruit setting period	BP	0.89	0.0212	3.17	0.88	0.0241	2.97
	PLSR	0.83	0.0265	2.54	0.81	0.0305	2.35
	LightGBM	0.93	0.017	3.95	0.85	0.0262	2.74
	1D-CNN	0.91	0.0191	3.52	0.84	0.0268	2.68
Fruit Enlargement Stage	BP	0.86	0.0211	2.67	0.83	0.0254	2.46
	PLSR	0.81	0.024	2.35	0.78	0.028	2.23
	LightGBM	0.90	0.018	3.13	0.82	0.026	2.4
	1D-CNN	0.88	0.0198	2.85	0.81	0.0265	2.36
Fruit Ripening Stage	BP	0.86	0.0203	2.7	0.85	0.0207	2.68
	PLSR	0.83	0.0221	2.48	0.81	0.0231	2.4
	LightGBM	0.92	0.015	3.66	0.86	0.0199	2.79
	1D-CNN	0.89	0.0182	3.01	0.80	0.0272	2.41

As presented in **Table 4**, during the fruit-setting period, the FD+CARS-BP model attained a Coefficient of determination (R²) of 0.88 on the validation set, surpassing PLSR (0.81) and 1D-CNN (0.84). Although its R² was marginally lower than that of LightGBM (0.85), the model demonstrated a lower Root mean square error (RMSE) (0.0241) and a higher RPD (2.97), indicating more stable and reliable prediction performance. During the fruit swelling period, the SG+FD-CARS-BP model achieved a validation set Coefficient of determination (R²) of 0.83 and an Root mean square error (RMSE) of 0.0254, outperforming both PLSR and 1D-CNN, and performing comparably to LightGBM. Notably, while LightGBM attained a high Coefficient of determination (R²) on the Training set (0.90), its performance on the validation set declined significantly (0.82), suggesting potential overfitting. In contrast, the model proposed in this study exhibited more consistent performance across both training and validation sets, indicating superior Generalization ability.

**Table 4:** Comparison of intertemporal models.

Model	Period	Training set			Validation set		
		R^2^	RMSEC	RPD	R^2^	RMSEC	RPD
Reproductive Stage MSC-CARS-BP Model	fruit setting stage	0.94	0.0174	4.0725	0.73	0.0350	1.9914
	Fruit Enlargement Stage	0.82	0.0248	2.3936	0.77	0.0258	2.3936
	Fruit Ripening Period	0.74	0.0280	2.0821	0.69	0.0286	1.8807
Fruit Setting Period FD+CARS-BP Model	fruit setting period	0.89	0.0212	3.1696	0.88	0.0241	2.9663
	Fruit Enlargement Stage	0.86	0.0264	2.8344	0.78	0.0323	2.4215
	Fruit Ripening Period	0.82	0.0245	2.3448	0.78	0.0270	2.1481
Fruit Swelling Stage SG+FD-CARS-BP Model	fruit-setting period	0.79	0.0262	2.2430	0.77	0.0285	2.3265
	Fruit Enlargement Stage	0.86	0.0211	2.6721	0.83	0.0254	2.4571
	Fruit Ripening Period	0.84	0.0224	2.6924	0.76	0.0285	2.1165
Fruit Ripening Stage SG+SD-CARS-BP Model	fruit setting stage	0.80	0.0246	2.2669	0.81	0.0280	2.2801
	Fruit Swelling Stage	0.70	0.0306	1.8600	0.73	0.0330	1.9688
	Fruit Ripening Period	0.86	0.0203	2.6977	0.85	0.0207	2.6844

At the Maturity period, the SG+SD-CARS-BP model delivered the best overall predictive performance, with a validation set Coefficient of determination (R²) of 0.85, an Root mean square error (RMSE) of 0.0207, and an RPD of 2.68. All metrics surpassed those of PLSR and 1D-CNN. Compared to LightGBM, the proposed model showed better performance in terms of Root mean square error (RMSE) and RPD, further highlighting its accuracy and stability in practical applications. In summary, systematic comparisons with multiple advanced baseline model demonstrate that the Growth period-specific machine learning model developed in this study exhibits consistently excellent and stable predictive ability across different growth stages, confirming the effectiveness and superiority of the Stage-based modeling strategy for monitoring LTP content in Korla fragrant pear leaves. 

## 4 Discussion

This study systematically investigates the prediction of Leaf total phosphorus (LTP) content in Korla Fragrant Pear, comprehensively revealing the application patterns of Near-Infrared Spectroscopy in fruit nutrition diagnosis through the analysis of Growth period differences, spectral pre-processing refinement, trait screening, and model construction and validation. It provides theoretical and technical support for Precision nutrient management in orchard. Specific discussions are as follows.

### 4.1 The dynamic of LTP content during the fertile period and model adaptability

Different growth stages Korla Fragrant Pear leaf LTP content showed significant differences (P< 0.05), with low and discrete content during the fruit-setting period, stable during the fruit expansion period, and high and discrete during the Maturity period (**Figure 2**). The FD+CARS-BP model performed excellently during the fruit-setting period, with R² of training sets reaching 0.90504 and validation set R² of 0.88785. FD preprocessing enhanced spectral dynamic differences, and the CARS algorithm accurately screening bands associated with highly discrete LTP, adapting to the “high dynamic phosphorus content-complex Spectral response” trait (Yu et al., 2024).;Fruit expansion period SG+FD-CARS-BP model leverages the synergistic effect of SG noise reduction and FD enhancement to balance the “Stable Spectra-Basic Phosphorus Absorption” relationship, with R2 values of 0.86243 and 0.83488 for training sets and validation set respectively; Maturity period SG+SD-CARS-BP model utilizes SG and SD fine feature extraction to effectively capture trace phosphorus association information, achieving R2 values of 0.87246 and 0.86146 for training sets and validation set respectively. This demonstrates that the Growth-period-specific model significantly improves prediction accuracy by adapting to the dynamics of LTP content across different periods (high dispersion, stable state, low concentration), validating the necessity of “Stage-based modeling” in fruit nutrition diagnosis. These findings align with the conclusions of Li et al (Bing zhi et al., 2010). in their study on hyperspectral estimation models of total nitrogen content in apple tree leaf leaves, which reflects the growth period adaptation law of fruit tree nutrition spectral diagnosis.

### 4.2 The synergistic mechanism of spectral preprocessing

Single preprocessing (MSC, SG, FD, SD) optimizes spectra from perspectives of physical interference elimination, noise suppression, dynamic enhancement, and fine feature extraction, yet exhibits functional limitations (e.g., FD tends to amplify noise, Second derivative is sensitive to noise) (Bao et al., 2024). Combined preprocessing (e.g., MS+FD, SG+SD achieves “multi-functional synergy”: MSC+FD first eliminates scattering interference and then amplifies chemical absorption dynamic change, enhancing the Spectral response of highly discrete LTP during the fruit-setting period; SG+SD first reduces noise to smooth the curve and then extracts fine structures of absorption peak, adapting to weak signals of low concentrations in the Maturity period. Correlation analysis shows that combined strategies can increase the r value of typical peaks and valleys by 0.05–0.15, demonstrating that the “synergistic effect” of preprocessing is key to extracting Phosphorus-association information, providing an effective approach for spectral refinement of complex samples. This aligns with the consensus in the Chemometrics field that “Combined preprocessing enhances Model performance”, clarifying the refinement direction of spectral pre-processing in orchard Phosphorus diagnosis (Li et al., 2024).

### 4.3 Optimization of model hyper parameter and its impact on model performance

The performance of a Machine learning model is significantly influenced by the selection of hyper parameter, and the refinement of these hyper parameter directly affects the model’s Generalization ability and Prediction accuracy (Schratz et al., 2019; Yang and Shami, 2020). Most existing studies have directly used default parameters to construct Spectroscopy estimation models without in-depth algorithmic refinement, which limits the performance improvement of the models. To address this limitation, this study systematically conducted research on hyper parameter refinement, employing grid search and cross validation methods to finely tune the parameters of Random forest (RF), Support vector machine (SVR), and BP neural network, significantly enhancing the stability and Prediction accuracy of the models.

For the RF model, experiments found that when n_estimators is 500 and min_samples_leaf is 5, the model achieves an optimal balance between training and prediction, effectively avoiding the phenomena of overfitting and poor fitting. In the Support Vector Regression model, the Penalty coefficient (C) C = 5 and kernel parameter γ=0.1 were determined, and the Radial Basis Function (RBF) was selected. The Model performance outperformed other configurations such as linear kernel and polynomial kernel.

In the BP neural network, by comparing various training functions, the trainlm function was ultimately identified as the most suitable for the research task, achieving an ideal Fitting effect while ensuring convergence speed.

The above optimization results indicate that conducting parameter optimization for different algorithm systems can effectively exploit the model’s potential and avoid the performance shortcomings caused by directly using default parameters. Through meticulous parameter tuning, this study provides a reliable configuration foundation for building a high-accuracy LTP content prediction model, and also offers a reference for algorithm optimization in similar Spectral modeling research.To ensure optimal Model performance, this study employed grid search and cross validation to refine the hyper parameters of RF, Support Vector Regression, and BP. Neither overfitting nor poor fitting phenomena occurred, further confirming the excellent performance of the model under this parameter combination. Therefore, the optimal parameters for this model are a number of decision trees of 500 and a min_samples_leaf of 5. Consequently, it is concluded that the model performs best when C = 5 and γ=0.1. To further establish the model, the results indicated that the performance of the Radial Basis Function (RBF) is superior to the other three functions. Under the results, it was found that the trainlm training function is more suitable for this model.

### 4.4 Comparison with advanced baseline models

In previous studies, Partial least squares regression (PLSR), Light Gradient Boosting Machine (LightGBM) (LightGBM), and One-dimensional convolutional neural network (1D-CNN) have been widely used in the field of Spectroscopy. For example, researchers such as those from AgResearch employed ryegrass as experimental material and constructed a Spectroscopy prediction model using PLSR to evaluate the composition of ryegrass plants. Their results demonstrated strong predictive performance for total polysaccharide (R² = 0.58), High molecular weight sugars (R² = 0.63), ash (R² = 0.50), and nitrogen content (R² = 0.70) (Shorten et al., 2019). In another study, Jun Yan et al. used maize to develop a LightGBM-based prediction model for genomic selection prediction of maize lines. The model achieved an Area Under the Curve (AUC) of 0.793, indicating excellent performance in classification tasks involving large sample sizes (Yan et al., 2021).Guo, C. et al. constructed a cotton Fv/Fm prediction model based on 1D-CNN for drought tolerance assessment, using cotton as the experimental material. The predicted value showed a strong Correlation with the measured value (R² ≥ 0.641). The results demonstrate that 1D-CNN offers high accuracy and stability in processing Large-scale data (Guo et al., 2022).

Although these models have shown excellent performance in studies by various researchers, the stability of PLSR under specific conditions across Different growth stages requires further enhancement. Both LightGBM and 1D-CNN are prone to high squared errors or significant bias when training samples are limited, increasing the risk of poor fitting. In comparison, this study identified more adaptive optimal Modeling strategies for Spectral features at Different growth stages through rigorous screening: the fruit-setting stage employs the FD+CARS-BP model, the expansion stage uses the SG+FD-CARS-BP model, and the Maturity period favors the SG+SD-CARS-BP model. The results show that these models exhibit superior predictive stability and adaptability across Different growth stages, enabling them to better handle the challenges of Modeling with small sample sizes, thus improving the accuracy of component prediction during specific growth stages.

### 4.5 Model generalization ability and cross-period challenges

Cross-period model comparisons revealed that the special models exhibited an R2 value 0.05–0.16 higher than that of the general model during this growth period, while the Root mean square error (RMSE) was 0.0029–0.0079 lower. Due to its inability to adapt to the “dynamic Spectroscopy fingerprint” of LTP across Different growth stages (such as the high discrete peak during the Fruit-setting period and the weak signal peak during the Maturity period), when the Fruit-setting period FD+CARS-BP model was extended to the Fruit expansion period, the R2 of the validation set decreased from 0.88 to 0.78, reflecting the specificity of the “Spectroscopy–phosphorus content” relationship across growth periods. In practical applications, it is necessary to switch models based on the growth period or explore intertemporal transfer learning strategies (such as fine-tuning parameters of a pre-trained model) to balance model accuracy and convenience. This provides practical references for the field application and Roll out of orchard Spectroscopy models and also clarifies the direction for future model refinement—enhancing the model’s adaptability to differences between growth periods.

### 4.6 Limitations of the study and future directions

This study focuses on near-infrared spectroscopy (4000–10000 cm^-^¹), with insufficient exploration of phosphorus characteristic peak (such as P–O bond stretching vibration, ~1000–1300 cm^-^¹). Future work could integrate Mid-infrared spectroscopy to expand features’ dimension, while simultaneously refining preprocessing and model parameter. Moreover, model training relies on laboratory Spectral data, without fully accounting for interference from field environments (e.g., light, temperature) on spectra. It is necessary to develop field spectral correction models and incorporate dynamic parameter adjustments to enhance technical practicality, thereby promoting the transition of Spectral diagnosis technology from the laboratory to practical application and improving the technical system for precision nutrient management in fruit trees.

In summary, this study clarifies the “Growth period specificity-Pretreatment synergy-model adaptation” technical framework for predicting Korla fragrant pear LTP, demonstrating that Stage-based modeling combined with Combined preprocessing can significantly improve prediction accuracy, providing a scientific paradigm for precision nutrient management in fruit trees. Subsequent efforts need to strengthen the integration of multiple Spectroscopy and field validation to further promote the application of Spectroscopy technology in orchard production.

## 5 Conclusion

This study systematically analyzed the Leaf total phosphorus (LTP) content of Korla Fragrant Pear using Near-Infrared Spectroscopy, established a prediction model based on Growth period characteristics, and significantly improved detection accuracy and model applicability. The main conclusions include:

The LTP content of Korla Fragrant Pear leaves showed significant differences across various growing stages. The content was lowest during the Fruit-setting period, with a left-skewed distribution ranging from 0.02% to 0.25%; it stabilized during the Fruit expansion period, with a median of approximately 0.15%; and peaked during the Maturity period, exhibiting a right-skewed distribution with a maximum value of 0.45%. spectral analysis revealed that Spectral features in the 4000–5500 cm^-^¹ and 5500–7500 cm^-^¹ ranges were closely correlated with phosphorus content, providing a basis for developing the prediction model. This study constructed an LTP prediction model adapted to Different growth stages. The optimal model for the fruit-setting period was FD+CARS-BP, with the Coefficient of determination (R²) for the training sets and validation set being 0.89 and 0.88, respectively; the optimal model for the fruit expansion period was SG+FD-CARS-BP, with the Coefficient of determination (R²) for the training sets and validation set being 0.86 and 0.83, respectively; the optimal model for the Maturity period was SG+SD-CARS-BP, with the Coefficient of determination (R²) for the training sets and validation set being 0.86 and 0.85, respectively. The predictive performance of all stage-specific models was significantly better than that of the intertemporal general model, with the Coefficient of determination (R²) increasing by 0.05–0.16 and the Root mean square error (RMSE) decreasing by 0.0029–0.0079. This has practical implications for precision fertilization management in orchards and provides a basis for subsequent research to further enrich the trait system by combining Mid-infrared spectroscopy technology and to develop calibration models for real field environments, thereby enhancing the practicality and roll-out value of the method.

*commercial or financial relationships that could be construed as a potential conflict of interest*.

## Data Availability

The original contributions presented in the study are included in the article/supplementary material. Further inquiries can be directed to the corresponding author.
