# Supplementary material for: Near-infrared prediction of total phosphorus in leaves content in korla fragrant pear with growth period specificity via spectral modeling
Source: Front Plant Sci. 2025 Oct 10;16:1666460. doi: 10.3389/fpls.2025.1666460 (PMC12549709; doi:10.3389/fpls.2025.1666460)
Supplement: Supplementary file 1 [file Table1.docx]

Table 1 Results of the whole growth period model

| Model | Training set | | | validation set | | |
| --- | --- | --- | --- | --- | --- | --- |
|  | R^2^ | RMSE | RPD | R^2^ | RMSE | RPD |
| MSC-CARS-RF | 0.7453 | 0.0337 | 1.9831 | 0.6844 | 0.0344 | 1.7801 |
| SG+CARS-RF | 0.7762 | 0.0234 | 2.0880 | 0.6668 | 0.0384 | 1.7638 |
| FD+CARS-RF | 0.7797 | 0.0296 | 2.1421 | 0.7424 | 0.0318 | 2.0003 |
| SD+CARS-RF | 0.1951 | 0.0579 | 1.2434 | 0.0284 | 0.0623 | 1.1508 |
| MSC+FD-CARS-RF | 0.9087 | 0.0193 | 3.3100 | 0.7128 | 0.0370 | 1.3118 |
| MSC+SD-CARS-RF | 0.7951 | 0.0269 | 2.2094 | 0.7611 | 0.0334 | 2.0521 |
| SG+FD-CARS-RF | 0.7889 | 0.0276 | 2.2441 | 0.7153 | 0.0320 | 1.9167 |
| SG+SD-CARS-RF | 0.7760 | 0.0282 | 2.1145 | 0.7004 | 0.0231 | 1.9039 |
| MSC-CARS-BP | 0.8367 | 0.0258 | 2.4436 | 0.8140 | 0.0268 | 2.3764 |
| SG+CARS-BP | 0.9412 | 0.0147 | 4.1281 | 0.8527 | 0.0198 | 2.9376 |
| FD+CARS-BP | 0.8908 | 0.0201 | 3.0704 | 0.8025 | 0.0229 | 2.3825 |
| SD+CARS-BP | 0.9056 | 0.0207 | 3.2669 | 0.3584 | 0.0364 | 1.2486 |
| MSC+FD-CARS-BP | 0.8979 | 0.0199 | 3.1487 | 0.8005 | 0.0254 | 2.2725 |
| MSC+SD-CARS-BP | 0.6767 | 0.0354 | 1.8006 | 0.5317 | 0.0389 | 1.6405 |
| SG+FD-CARS-BP | 0.8607 | 0.0233 | 2.6808 | 0.7952 | 0.0257 | 2.2341 |
| SG+SD-CARS-BP | 0.8855 | 0.0211 | 2.9566 | 0.7227 | 0.0299 | 2.0794 |
| MSC-CARS-SVM | 0.8698 | 0.0227 | 3.7888 | 0.7953 | 0.0299 | 1.9988 |
| SG+CARS-SVM | 0.8379 | 0.0203 | 2.1825 | 0.7387 | 0.0316 | 1.0860 |
| FD+CARS-SVM | 0.9791 | 0.0095 | 10.3047 | 0.8146 | 0.0250 | 2.3454 |
| SD+CARS-SVM | 0.8016 | 0.0299 | 1.7076 | 0.7997 | 0.0288 | 1.7431 |
| MSC+FD-CARS-SVM | 0.7935 | 0.0287 | 2.1862 | 0.7781 | 0.0290 | 2.0917 |
| MSC+SD-CARS-SVM | 0.7578 | 0.0291 | 1.4881 | 0.6998 | 0.0344 | 1.3034 |
| SG+FD-CARS-SVM | 0.8149 | 0.0279 | 2.3482 | 0.8441 | 0.0249 | 2.5449 |
| SG+SD-CARS-SVM | 0.7826 | 0.0289 | 1.8462 | 0.7887 | 0.0286 | 1.9136 |

Table 2 Specific model results of fruit setting period

| Model | Training set | | | validation set | | |
| --- | --- | --- | --- | --- | --- | --- |
|  | R^2^ | RMSE | RPD | R^2^ | RMSE | RPD |
| MSC-CARS-RF | 0.2029 | 0.0543 | 1.2021 | 0.2111 | 0.0592 | 1.2766 |
| SG+CARS-RF | 0.9130 | 0.0202 | 3.3909 | 0.7178 | 0.0361 | 1.8832 |
| FD+CARS-RF | 0.8178 | 0.0265 | 2.4585 | 0.7546 | 0.0323 | 2.0481 |
| SD+CARS-RF | 0.7600 | 0.0323 | 2.0540 | 0.7397 | 0.0330 | 1.9637 |
| MSC+FD-CARS-RF | 0.8745 | 0.0260 | 2.9030 | 0.7789 | 0.0297 | 2.1405 |
| MSC+SD-CARS-RF | 0.6945 | 0.0328 | 1.8115 | 0.6830 | 0.0388 | 1.8420 |
| SG+FD-CARS-RF | 0.8482 | 0.0267 | 2.5807 | 0.7725 | 0.0295 | 2.0965 |
| SG+SD-CARS-RF | 0.7887 | 0.0329 | 2.2010 | 0.7707 | 0.0376 | 2.1147 |
| MSC-CARS-BP | 0.6599 | 0.0606 | 1.7567 | 0.1373 | 0.0433 | 0.8470 |
| SG+CARS-BP | 0.9814 | 0.0065 | 7.7902 | 0.7624 | 0.0137 | 2.8954 |
| FD+CARS-BP | 0.8938 | 0.0212 | 3.1696 | 0.8849 | 0.0241 | 2.9663 |
| SD+CARS-BP | 0.8889 | 0.0227 | 3.0055 | 0.7544 | 0.0353 | 2.0478 |
| MSC+FD-CARS-BP | 0.8555 | 0.0273 | 2.6584 | 0.8512 | 0.0244 | 2.5932 |
| MSC+SD-CARS-BP | 0.9637 | 0.0132 | 5.2830 | 0.8327 | 0.0283 | 0.8327 |
| SG+FD-CARS-BP | 0.8840 | 0.0233 | 2.9524 | 0.8101 | 0.0312 | 2.2949 |
| SG+SD-CARS-BP | 0.8778 | 0.0247 | 3.2125 | 0.7422 | 0.0331 | 2.0778 |
| MSC-CARS-SVM | 0.8878 | 0.0230 | 4.2332 | 0.7827 | 0.0328 | 1.8186 |
| SG+CARS-SVM | 0.7930 | 0.0318 | 1.8189 | 0.7848 | 0.0324 | 1.7933 |
| FD+CARS-SVM | 0.7390 | 0.0359 | 1.8022 | 0.8029 | 0.0284 | 1.9682 |
| SD+CARS-SVM | 0.7792 | 0.0323 | 1.8692 | 0.8060 | 0.0348 | 1.7685 |
| MSC+FD-CARS-SVM | 0.8060 | 0.0242 | 2.4226 | 0.7892 | 0.0321 | 1.7760 |
| MSC+SD-CARS-SVM | 0.8397 | 0.0279 | 2.2024 | 0.7873 | 0.0281 | 1.9787 |
| SG+FD-CARS-SVM | 0.8417 | 0.0297 | 2.3657 | 0.6686 | 0.0370 | 1.7139 |
| SG+SD-CARS-SVM | 0.8141 | 0.0319 | 1.9064 | 0.7811 | 0.0325 | 1.8651 |

Table 3 Specific model results of fruit expansion period

| Model | Training set | | | validation set | | |
| --- | --- | --- | --- | --- | --- | --- |
|  | R^2^ | RMSE | RPD | R^2^ | RMSE | RPD |
| MSC-CARS-RF | 0.7158 | 0.0367 | 1.8897 | 0.7182 | 0.0354 | 2.0472 |
| SG+CARS-RF | 0.2475 | 0.0440 | 1.0008 | 0.2486 | 0.0403 | 1.0013 |
| FD+CARS-RF | 0.7085 | 0.0290 | 1.8529 | 0.7165 | 0.0320 | 1.9304 |
| SD+CARS-RF | 0.7787 | 0.0281 | 2.1289 | 0.6856 | 0.0332 | 1.8717 |
| MSC+FD-CARS-RF | 0.7938 | 0.0278 | 2.2217 | 0.7385 | 0.0305 | 1.9620 |
| MSC+SD-CARS-RF | 0.7988 | 0.0257 | 2.2293 | 0.8171 | 0.0238 | 2.3391 |
| SG+FD-CARS-RF | 0.8836 | 0.0209 | 2.9314 | 0.7334 | 0.0287 | 1.9420 |
| SG+SD-CARS-RF | 0.7251 | 0.0326 | 1.9508 | 0.7188 | 0.0336 | 1.8881 |
| MSC-CARS-BP | 0.8351 | 0.0238 | 2.4657 | 0.8161 | 0.0244 | 2.3335 |
| SG+CARS-BP | 0.4534 | 0.1377 | 1.4287 | 0.3501 | 0.1469 | 1.3369 |
| FD+CARS-BP | 0.7833 | 0.0239 | 2.2264 | 0.7642 | 0.0267 | 2.0613 |
| SD+CARS-BP | 0.8856 | 0.0200 | 2.9627 | 0.5656 | 0.0323 | 1.5339 |
| MSC+FD-CARS-BP | 0.7703 | 0.0297 | 2.9510 | 0.5917 | 0.0290 | 1.8286 |
| MSC+SD-CARS-BP | 0.7692 | 0.0263 | 2.0927 | 0.6210 | 0.0386 | 1.6253 |
| SG+FD-CARS-BP | 0.8594 | 0.0211 | 2.6721 | 0.8331 | 0.0254 | 2.4571 |
| SG+SD-CARS-BP | 0.8775 | 0.0200 | 2.8603 | 0.7105 | 0.0313 | 1.9749 |
| MSC-CARS-SVM | 0.8951 | 0.0194 | 4.3079 | 0.8081 | 0.0228 | 2.2185 |
| SG+CARS-SVM | 0.7994 | 0.0243 | 1.8983 | 0.7876 | 0.0271 | 1.7903 |
| FD+CARS-SVM | 0.8639 | 0.0216 | 2.2363 | 0.7926 | 0.0247 | 1.8586 |
| SD+CARS-SVM | 0.4448 | 0.0413 | 0.4836 | 0.3699 | 0.0470 | 0.3829 |
| MSC+FD-CARS-SVM | 0.7332 | 0.0252 | 1.2541 | 0.7525 | 0.0283 | 1.4643 |
| MSC+SD-CARS-SVM | 0.6888 | 0.0301 | 0.9900 | 0.5492 | 0.0383 | 0.7050 |
| SG+FD-CARS-SVM | 0.9717 | 0.0094 | 8.9819 | 0.8357 | 0.0249 | 2.2149 |
| SG+SD-CARS-SVM | 0.7811 | 0.0263 | 1.4237 | 0.7068 | 0.0294 | 1.2966 |

Table 4 Fruit maturity-specific model results

| Model | Training set | | | validation set | | |
| --- | --- | --- | --- | --- | --- | --- |
|  | R2 | RMSE | RPD | R2 | RMSE | RPD |
| MSC-CARS-RF | 0.8156 | 0.0289 | 2.3432 | 0.7803 | 0.0302 | 2.2025 |
| SG+CARS-RF | 0.8951 | 0.0221 | 3.0874 | 0.7449 | 0.0358 | 1.9863 |
| FD+CARS-RF | 0.7772 | 0.0234 | 2.1329 | 0.6113 | 0.0308 | 1.6696 |
| SD+CARS-RF | 0.7851 | 0.0249 | 2.1574 | 0.6965 | 0.0287 | 1.8253 |
| MSC+FD-CARS-RF | 0.7192 | 0.0298 | 1.9906 | 0.6983 | 0.0257 | 1.8502 |
| MSC+SD-CARS-RF | 0.7531 | 0.0273 | 2.0187 | 0.7413 | 0.0275 | 1.9665 |
| SG+FD-CARS-RF | 0.8129 | 0.0231 | 2.3696 | 0.8017 | 0.0237 | 2.2457 |
| SG+SD-CARS-RF | 0.6914 | 0.0309 | 1.8368 | 0.7098 | 0.0281 | 1.9440 |
| MSC-CARS-BP | 0.7802 | 0.0268 | 2.1880 | 0.6468 | 0.0300 | 1.7838 |
| SG+CARS-BP | 0.6273 | 0.0345 | 1.6561 | 0.6950 | 0.0310 | 1.6886 |
| FD+CARS-BP | 0.8884 | 0.0188 | 2.9929 | 0.7432 | 0.0258 | 1.9865 |
| SD+CARS-BP | 0.7715 | 0.0251 | 2.1071 | 0.7623 | 0.0289 | 2.0864 |
| MSC+FD-CARS-BP | 0.8586 | 0.0206 | 2.7606 | 0.6362 | 0.0316 | 1.8572 |
| MSC+SD-CARS-BP | 0.8886 | 0.0177 | 3.0047 | 0.7388 | 0.0292 | 1.9574 |
| SG+FD-CARS-BP | 0.8371 | 0.0217 | 2.4852 | 0.7117 | 0.0298 | 1.8661 |
| SG+SD-CARS-BP | 0.8625 | 0.0203 | 2.6977 | 0.8549 | 0.0207 | 2.6844 |
| MSC-CARS-SVM | 0.8185 | 0.0227 | 3.0751 | 0.8552 | 0.0218 | 2.2088 |
| SG+CARS-SVM | 0.8299 | 0.0216 | 3.2851 | 0.8006 | 0.0267 | 1.8769 |
| FD+CARS-SVM | 0.8357 | 0.0234 | 2.2030 | 0.7711 | 0.0212 | 1.8978 |
| SD+CARS-SVM | 0.7088 | 0.0274 | 1.0935 | 0.7190 | 0.0256 | 1.1946 |
| MSC+FD-CARS-SVM | 0.7603 | 0.0306 | 1.4803 | 0.6707 | 0.0300 | 1.3648 |
| MSC+SD-CARS-SVM | 0.5129 | 0.0394 | 0.6196 | 0.5256 | 0.0372 | 0.6135 |
| SG+FD-CARS-SVM | 0.9675 | 0.0100 | 8.1236 | 0.8020 | 0.0232 | 2.0949 |
| SG+SD-CARS-SVM | 0.7869 | 0.0297 | 1.8476 | 0.5025 | 0.0374 | 1.3548 |
